# Association of Chromosome 9p21 With Subsequent Coronary Heart Disease Events: A GENIUS-CHD Study of Individual Participant Data

**DOI:** 10.1161/CIRCGEN.119.002471

**Published:** 2019-04-16

**Authors:** Riyaz S. Patel, Amand F. Schmidt, Vinicius Tragante, Raymond O. McCubrey, Michael V. Holmes, Laurence J. Howe, Kenan Direk, Axel Åkerblom, Karin Leander, Salim S. Virani, Karol A. Kaminski, Jochen D. Muehlschlegel, Marie-Pierre Dubé, Hooman Allayee, Peter Almgren, Maris Alver, Ekaterina V. Baranova, Hassan Behlouli, Bram Boeckx, Peter S. Braund, Lutz P. Breitling, Graciela Delgado, Nubia E. Duarte, Line Dufresne, Niclas Eriksson, Luisa Foco, Crystel M. Gijsberts, Yan Gong, Jaana Hartiala, Mahyar Heydarpour, Jaroslav A. Hubacek, Marcus Kleber, Daniel Kofink, Pekka Kuukasjärvi, Vei-Vei Lee, Andreas Leiherer, Petra A. Lenzini, Daniel Levin, Leo-Pekka Lyytikäinen, Nicola Martinelli, Ute Mons, Christopher P. Nelson, Kjell Nikus, Anna P. Pilbrow, Rafal Ploski, Yan V. Sun, Michael W.T. Tanck, W.H.Wilson Tang, Stella Trompet, Sander W. van der Laan, Jessica van Setten, Ragnar O. Vilmundarson, Chiara Viviani Anselmi, Efthymia Vlachopoulou, Eric Boerwinkle, Carlo Briguori, John F. Carlquist, Kathryn F. Carruthers, Gavino Casu, John Deanfield, Panos Deloukas, Frank Dudbridge, Natalie Fitzpatrick, Bruna Gigante, Stefan James, Marja-Liisa Lokki, Paulo A. Lotufo, Nicola Marziliano, Ify R. Mordi, Joseph B. Muhlestein, Chris Newton Cheh, Jan Pitha, Christoph H. Saely, Ayman Samman-Tahhan, Pratik B. Sandesara, Andrej Teren, Adam Timmis, Frans Van de Werf, Els Wauters, Arthur A.M. Wilde, Ian Ford, David J. Stott, Ale Algra, Maria G. Andreassi, Diego Ardissino, Benoit J. Arsenault, Christie M. Ballantyne, Thomas O. Bergmeijer, Connie R. Bezzina, Simon C. Body, Peter Bogaty, Gert J. de Borst, Hermann Brenner, Ralph Burkhardt, Clara Carpeggiani, Gianluigi Condorelli, Rhonda M. Cooper-DeHoff, Sharon Cresci, Ulf de Faire, Robert N. Doughty, Heinz Drexel, James C. Engert, Keith A.A. Fox, Domenico Girelli, Emil Hagström, Stanley L. Hazen, Claes Held, Harry Hemingway, Imo E. Hoefer, G. Kees Hovingh, Julie A. Johnson, Pim A. de Jong, J. Wouter Jukema, Marcin P. Kaczor, Mika Kähönen, Jiri Kettner, Marek Kiliszek, Olaf H. Klungel, Bo Lagerqvist, Diether Lambrechts, Jari O. Laurikka, Terho Lehtimäki, Daniel Lindholm, Bakhtawar K. Mahmoodi, Anke H. Maitland-van der Zee, Ruth McPherson, Olle Melander, Andres Metspalu, Witold Pepinski, Oliviero Olivieri, Grzegorz Opolski, Colin N. Palmer, Gerard Pasterkamp, Carl J. Pepine, Alexandre C. Pereira, Louise Pilote, Arshed A. Quyyumi, A. Mark Richards, Marek Sanak, Markus Scholz, Agneta Siegbahn, Juha Sinisalo, J. Gustav Smith, John A. Spertus, Alexandre F.R. Stewart, Wojciech Szczeklik, Anna Szpakowicz, Jurriën M. ten Berg, George Thanassoulis, Joachim Thiery, Yolanda van der Graaf, Frank L.J. Visseren, Johannes Waltenberger, CARDIoGRAMPlusC4D Consortium, Pim Van der Harst, Jean-Claude Tardif, Naveed Sattar, Chim C. Lang, Guillaume Pare, James M. Brophy, Jeffrey L. Anderson, Winfried März, Lars Wallentin, Vicky A. Cameron, Benjamin D. Horne, Nilesh J. Samani, Aroon D. Hingorani, Folkert W. Asselbergs

**Affiliations:** 1 Institute of Cardiovascular Science, Faculty of Population Health Science (R.S.P., A.F.S., L.J.H., K.D., J.D., A.D.H., F.W.A.); 2 Institute of Health Informatics, Faculty of Population Health Science, University College London, United Kingdom (N.F., C.H.S., A. Timmis, H.H., F.W.A.).; 3 Bart’s Heart Centre, St Bartholomew’s Hospital, London, United Kingdom (R.S.P., J.D., A. Timmis).; 4 Division Heart and Lungs, Department of Cardiology (A.F.S., V.T. D.K., F.W.A.); 5 Laboratory of Experimental Cardiology (C.M.G., B.D.H.); 6 Department of Clinical Chemistry and Hematology (B.G., I.E.H.); 7 Department of Clinical Chemistry, UMC Utrecht, Netherlands (G. Pasterkamp).; 8 Intermountain Heart Institute, Intermountain Medical Center, Salt Lake City, UT (R.O.M., J.F.C., J.B.M., J.L.A.).; 9 Clinical Trial Service Unit and Epidemiological Studies Unit, Nuffield Department of Population Health (M.V.H.), University of Oxford, United Kingdom.; 10 Medical Research Council Population Health Research Unit (M.V.H.), University of Oxford, United Kingdom.; 11 National Institute for Health Research Oxford Biomedical Research Centre (M.V.H.), University of Oxford, United Kingdom.; 12 Uppsala Clinical Research Center (A.A., N.E., S.J., E.H., C.H., B.L., D. Lindholm, A. Siegbahn, L.W.), Uppsala University, Sweden.; 13 Department of Medical Sciences, Cardiology (A.A., E.H., C.H., D. Lindholm), Uppsala University, Sweden.; 14 Department of Medical Sciences, Cardiology (S.J., B.L., L.W.), Uppsala University, Sweden.; 15 Department of Medical Sciences, Clinical Chemistry (A. Siegbahn), Uppsala University, Sweden.; 16 Institute of Environmental Medicine, Karolinska Institutet, Stockholm, Sweden (K.L., U.d.F.).; 17 Section of Cardiology, Michael E. DeBakey Veterans Affairs Medical Center, Section of Cardiovascular Research, and Department of Medicine, Baylor College of Medicine, Houston, TX (S.S.V., C.M.B.).; 18 Department of Population Medicine and Civilization Disease Prevention (K.A.K.); 19 Department of Cardiology (K.A.K., A. Szpakowicz); 20 Department of Forensic Medicine, Medical University of Bialystok, Poland (W.P., G.T.).; 21 Department of Anesthesiology, Perioperative and Pain Medicine, Brigham and Women’s Hospital (M.H.); 22 Harvard Medical School, Boston, MA (J.D.M., M.H. S.C.B.).; 23 Montreal Heart Institute (J.-C.T.); 24 Faculty of Medicine (J.-C.T.); 25 Université de Montréal, QC, Canada (M.-P.D.).; 26 Departments of Preventive Medicine and Biochemistry and Molecular Medicine (H.A., J.H.), Keck School of Medicine of USC, Los Angeles, CA.; 27 Institute for Genetic Medicine (J.H.), Keck School of Medicine of USC, Los Angeles, CA.; 28 Department of Clinical Sciences, Lund University, Malmö, Sweden (P.A., O.M.).; 29 Estonian Genome Center, Institute of Genomics (A.M.); 30 Department of Biotechnology, Institute of Molecular and Cell Biology, University of Tartu, Estonia (M.A., A.M.).; 31 Division of Pharmacoepidemiology and Clinical Pharmacology (E.V.B., O.H.K., A.H.M.-v.d.Z.), University Medical Center Utrecht, the Netherlands.; 32 Department of Neurology and Neurosurgery, Brain Centre Rudolf Magnus and Julius Center for Health Sciences and Primary Care (A. Algra), University Medical Center Utrecht, the Netherlands.; 33 Department of Radiology (P.A.d.J.), University Medical Center Utrecht, the Netherlands.; 34 Julius Center for Health Sciences and Primary Care (Y.v.d.G.), University Medical Center Utrecht, the Netherlands.; 35 Department of Vascular Medicine, University Medical Center Utrecht and Utrecht University, the Netherlands (F.L.J.V.).; 36 Centre for Outcomes Research and Evaluation, Research Institute of the McGill University Health Centre (H.B., L.D., L.P., J.M.B.).; 37 Research Institute of the McGill University Health Centre (J.C.E.).; 38 Division of Cardiology, Department of Medicine, Royal Victoria Hospital (J.C.E., G.T.); 39 Department of Medicine (L.P., J.M.B.); 40 Preventive and Genomic Cardiology, McGill University Health Centre, Montreal, QC, Canada (L.D., J.C.E., G.T.).; 41 Laboratory for Translational Genetics, Department of Human Genetics (B.B., D. Lambrechts); 42 Departement of Cardiovascular Sciences, KU Leuven, Belgium (F.V.d.W.).; 43 Laboratory for Translational Genetics, VIB Center for Cancer Biology, VIB, Belgium (B.B., D. Lambrechts).; 44 Department of Cardiovascular Sciences (P.S.B., C.P.N., N.J.S.) and Department of Health Sciences, University of Leicester, United Kingdom.; 45 BHF Cardiovascular Research Centre (F.D.), Glenfield Hospital, Leicester, United Kingdom.; 46 National Institute of Health Research (NIHR) Leicester Biomedical Research Centre (P.S.B., C.P.N.), Glenfield Hospital, Leicester, United Kingdom.; 47 Division of Clinical Epidemiology and Aging Research, German Cancer Research Center (DKFZ), Heidelberg (L.P.B., U.M., H.B.).; 48 Vth Department of Medicine, Medical Faculty Mannheim, Heidelberg University, Mannheim, Germany (G.D., M. Kleber, W.M.).; 49 Heart Institute, University of Sao Paulo, Brazil (N.E.D., A.C.P.).; 50 Institute for Biomedicine, Eurac Research, Affiliated Institute of the University of Lübeck, Bolzano, Italy (L.F.).; 51 Department of Pharmacotherapy and Translational Research and Center for Pharmacogenomics (Y.G., R.M.C.-D., J.A.J.); 52 Division of Cardiovascular Medicine, College of Medicine, University of Florida (J.A.J., C.J.P.).; 53 Centre for Experimental Medicine, Institut for Clinical and Experimental Medicine, Prague, Czech Republic (J.A.H., J.P.).; 54 Department of Cardio-Thoracic Surgery (P.K.); 55 Department of Clinical Chemistry (L.-P.L., T.L.); 56 Department of Cardiology (K.N.); 57 Department of Clinical Physiology (M. Kähönen); 58 Department of Cardio-Thoracic Surgery, Finnish Cardiovascular Research Center, Faculty of Medicine and Life Sciences, University of Tampere (J.O.L.).; 59 Department of Biostatistics and Epidemiology, Texas Heart Institute, Houston (V.-V.L.).; 60 Vorarlberg Institute for Vascular Investigation and Treatment (VIVIT), Feldkirch, Austria (A.L., C.H.S., H.D.).; 61 Private University of the Principality of Liechtenstein, Triesen, Liechtenstein (A.L., C.H.S., H.D.).; 62 Medical Central Laboratories, Feldkirch, Austria (A.L.).; 63 Department of Genetics, Statistical Genomics Division (P.A.L., S.C.); 64 Department of Medicine, Cardiovascular Division Washington University School of Medicine, St Louis, MO (S.C.).; 65 Division of Molecular and Clinical Medicine, School of Medicine, University of Dundee, Scotland, United Kingdom (D. Levin, I.R.M., C.C.L.).; 66 Department of Clinical Chemistry, Fimlab Laboratories, Tampere, Finland (L.-P.L., T.L.).; 67 Department of Medicine, University of Verona, Italy (N. Martinelli, D.G., O.O.).; 68 Department of Cardiology, Heart Center (K.N.); 69 Department of Clinical Physiology (M. Kähönen); 70 Department of Cardio-Thoracic Surgery, Heart Center, Tampere University Hospital, Finland (J.O.L).; 71 The Christchurch Heart Institute, University of Otago Christchurch, New Zealand (A.P.B., A.M.R., V.A.C.).; 72 Department of Medical Genetics (R.P.); 73 Department of Cardiology, Medical University of Warsaw, Poland (G.O.).; 74 Department of Epidemiology, Emory University Rollins School of Public Health (Y.V.S.); 75 Department of Biomedical Informatics (Y.V.S.); 76 Division of Cardiology, Department of Medicine, Emory Clinical Cardiovascular Research Institute, Emory University School of Medicine, Atlanta, GA (A.S.-T., P.B.S., A.A.Q.).; 77 Clinical Epidemiology and Biostatistics (M.W.T.T.); 78 AMC Heart Center (A.A.M.W., C.R.B.); 79 Clinical and Experimental Cardiology, Amsterdam Cardiovascular Sciences, Amsterdam UMC, Department of Respiratory Medicine, Academic Medical Center, University of Amsterdam, the Netherlands (A.H.M.-v.d.Z.).; 80 Department of Cellular and Molecular Medicine, Lerner Research Institute (W.H.W.T., S.L.H.); 81 Department of Cardiovascular Medicine, Heart and Vascular Institute and Center for Clinical Genomics (W.H.W.T.); 82 Department of Cardiovascular Medicine, Heart and Vascular Institute and Center for Microbiome and Human Health, Cleveland Clinic, OH (S.L.H.).; 83 Section of Gerontology and Geriatrics, Department of Internal Medicine (S.T.), Leiden University Medical Center.; 84 Department of Cardiology (S.T., J.W.J.), Leiden University Medical Center.; 85 Laboratory of Clinical Chemistry and Hematology, Division Laboratories, Pharmacy, and Biomedical Genetics (S.W.v.d.L.); 86 Department of Vascular Surgery, University Medical Center Utrecht, University Utrecht, the Netherlands (G.J.d.B.).; 87 Durrer Centre of Cardiogenetic Research, ICIN-Netherlands Heart Institute, Netherlands (J.v.S., F.W.B.).; 88 Ruddy Canadian Cardiovascular Genetics Centre (R.O.V., A.F.R.S.); 89 University of Ottawa Heart Institute (R.M.); 90 Department of Biochemistry, Microbiology and Immunology (R.O.V., A.F.R.S.); 91 Departments of Medicine and Biochemistry, Microbiology and Immunology, University of Ottawa, ON, Canada (R.M.).; 92 Department of Cardiovascular Medicine, Humanitas Clinical and Research Center, Milan, Italy (C.V.A., G.C).; 93 Transplantation Laboratory, Medicum (E.V., M.-L.L.); 94 Heart and Lung Center, Helsinki University Hospital University of Helsinki, Finland (J.S.).; 95 University of Texas School of Public Health, Houston (E.B.).; 96 Clinica Mediterranea, Naples, Italy (C.B.).; 97 Cardiology Division, Department of Internal Medicine (J.F.C., J.B.M., J.L.A.); 98 Department of Biomedical Informatics, University of Utah, Salt Lake City (B.D.H.).; 99 Cardiovascular Sciences (K.F.C.), University of Edinburgh.; 100 Emeritus Professor of Cardiology (K.A.A.F.), University of Edinburgh.; 101 ATS Sardegna, ASL 3, Nuoro (G. Casu, N. Marziliano).; 102 William Harvey Research Institute, Barts and the London Medical School (P.D.), Queen Mary University of London.; 103 Centre for Genomic Health (P.D.), Queen Mary University of London.; 104 Centro de Pesquisa Clinica, Hospital Universitario, Universidade de Sao Paulo, Brazil (P.A.L.).; 105 Cardiovascular Research Center and Center for Human Genetic Research, Massachusetts General Hospital, Boston and Program in Medical and Population Genetics, Broad Institute, Cambridge, MA (C.N.C.).; 106 Department of Medicine and Cardiology, Academic Teaching Hospital Feldkirch, Austria. Heart Center Leipzig (A. Teren); 107 LIFE Research Center for Civilization Diseases (A. Teren, R.B., M. Scholz, J.T.); 108 Institute for Medical Informatics, Statistics and Epidemiology, University of Leipzig, Germany (M. Scholz).; 109 Respiratory Oncology Unit, Department of Respiratory Medicine, University Hospitals KU Leuven, Belgium (E.W.).; 110 Princess Al-Jawhara Al-Brahim Centre of Excellence in Research of Hereditary Disorders, Jeddah, Saudi Arabia (A.A.M.W.).; 111 Robertson Center for Biostatistics (I.F.); 112 Institute of Cardiovascular and Medical Sciences, University of Glasgow, United Kingdom (D.J.S., N.S.).; 113 CNR Institute of Clinical Physiology, Pisa, Italy (M.G.A., C.C.).; 114 Cardiology Department, Parma University Hospital, Italy (D.A.).; 115 Centre de recherche de l’Institut Universitaire de cardiologie et de pneumologie de Québec (B.J.A.); 116 Department of Medicine, Faculty of Medicine, Université Laval, Canada (B.J.A.).; 117 St. Antonius Hospital, Department of Cardiology, Nieuwegein, the Netherlands (T.O.B., B.K.M., J.M.t.B.).; 118 Department of Anesthesia, Pain and Critical Care, Beth Israel Deaconess Medical Center, Boston, MA (S.C.B.).; 119 Service de cardiologie, Département multidisciplinaire de cardiologie, Instituteitut universitaire de cardiologie et de pneumologie de Québec, Canada (P.B.).; 120 Unité d’évaluation cardiovasculaire, Institut national d’excellence en santé et en services sociaux (INESSS), Montreal Canada (P.B.).; 121 Instituteitut universitaire de cardiologie et de pneumologie de Québec, Laval University, Québec City, Canada (P.B.).; 122 Institute of Clinical Chemistry and Laboratory Medicine, University Hospital Regensburg, Germany (R.B.).; 123 Department of Biomedical Sciences, Humanitas University, Milan, Italy (G. Condorelli).; 124 Heart Health Research Group, University of Auckland, New Zealand (R.N.D.).; 125 Drexel University College of Medicine, Philadelphia, PA (H.D.).; 126 Department of Vascular Medicine, Academic Medical Center, Amsterdam, the Netherlands (G.K.H.).; 127 Einthoven Laboratory for Experimental Vascular Medicine, LUMC, Leiden (J.W.J.).; 128 Interuniversity Cardiology Institute of the Netherlands, Utrecht, the Netherlands (J.W.J.).; 129 Department of Internal Medicine, Jagiellonian University Medical College, Kraków, Poland (M.P.K., M. Sanak, W.S.).; 130 Cardiology Centre, Institute for Clinical and Experimental Medicine, Prague, Czech Republic (J.K.).; 131 Department of Cardiology and Internal Diseases, Military Institute of Medicine, Warsaw, Poland (M. Kiliszek).; 132 Department of Internal Medicine, Skåne University Hospital, Malmö, Sweden (O.M.).; 133 Pat Macpherson Centre for Pharmacogenetics and Pharmacogenomics, Division of Molecular and Clinical Medicine, Ninewells Hospital and Medical School, Dundee (C.N.P.).; 134 Cardiovascular Research Institute, National University of Singapore (A.M.R.).; 135 Department of Cardiology, Clinical Sciences, Lund University and Skåne University Hospital (J.G.S.), Lund University, Lund, Sweden.; 136 Wallenberg Center for Molecular Medicine (J.G.S.), Lund University, Lund, Sweden.; 137 Lund University Diabetes Center (J.G.S.), Lund University, Lund, Sweden.; 138 Saint Luke’s Mid America Heart Institute and the University of Missouri-Kansas City and Saint Luke’s Health System, Kansas City, MO (J.A.S.).; 139 Institute of Laboratory Medicine, Clinical Chemistry and Molecular Diagnostics, University Hospital, Leipzig (J.T.).; 140 Department of Cardiovascular Medicine, University of Münster, Germany (J.W.).; 141 CARDIoGRAMPlusC4D. University of Groningen, University Medical Center, Groningen, Netherlands (P.V.d.H.).; 142 Department of Pathology and Molecular Medicine, McMaster University (G. Pare).; 143 Population Health Research Institute, Hamilton, ON, Canada (G. Pare).; 144 Synlab Academy, Synlab Holding Deutschland GmbH, Mannheim, Germany (W.M.).; 145 Clinical Institute of Medical and Chemical Laboratory Diagnostics, Medical University of Graz, Austria (W.M.).

**Keywords:** chromosome, genetic, variation, myocardial infarction, risk factor, secondary prevention

## Abstract

**Background:**

Genetic variation at chromosome 9p21 is a recognized risk factor for coronary heart disease (CHD). However, its effect on disease progression and subsequent events is unclear, raising questions about its value for stratification of residual risk.

**Methods:**

A variant at chromosome 9p21 (rs1333049) was tested for association with subsequent events during follow-up in 103 357 Europeans with established CHD at baseline from the GENIUS-CHD (Genetics of Subsequent Coronary Heart Disease) Consortium (73.1% male, mean age 62.9 years). The primary outcome, subsequent CHD death or myocardial infarction (CHD death/myocardial infarction), occurred in 13 040 of the 93 115 participants with available outcome data. Effect estimates were compared with case/control risk obtained from the CARDIoGRAMplusC4D consortium (Coronary Artery Disease Genome-wide Replication and Meta-analysis [CARDIoGRAM] plus The Coronary Artery Disease [C4D] Genetics) including 47 222 CHD cases and 122 264 controls free of CHD.

**Results:**

Meta-analyses revealed no significant association between chromosome 9p21 and the primary outcome of CHD death/myocardial infarction among those with established CHD at baseline (GENIUS-CHD odds ratio, 1.02; 95% CI, 0.99–1.05). This contrasted with a strong association in CARDIoGRAMPlusC4D odds ratio 1.20; 95% CI, 1.18–1.22; *P* for interaction <0.001 compared with the GENIUS-CHD estimate. Similarly, no clear associations were identified for additional subsequent outcomes, including all-cause death, although we found a modest positive association between chromosome 9p21 and subsequent revascularization (odds ratio, 1.07; 95% CI, 1.04–1.09).

**Conclusions:**

In contrast to studies comparing individuals with CHD to disease-free controls, we found no clear association between genetic variation at chromosome 9p21 and risk of subsequent acute CHD events when all individuals had CHD at baseline. However, the association with subsequent revascularization may support the postulated mechanism of chromosome 9p21 for promoting atheroma development.

## Abstract 2

Supplemental Digital Content is available in the text.

Using a case-control approach, a large number of common genetic variants have now been associated with coronary heart disease (CHD) through genome-wide association studies, in an effort largely led by the CARDIoGRAMPlusC4D consortium (Coronary Artery Disease Genome-wide Replication and Meta-analysis [CARDIoGRAM] plus The Coronary Artery Disease [C4D] Genetics).^1^ Among these variants, the chromosome 9p21 locus was the first to be discovered and the variant with the largest individual effect and is the most widely replicated genetic risk factor for CHD.^2–4^ Multiple studies including case-control and prospective cohort studies in general populations have reliably confirmed its effect on risk of CHD among otherwise healthy individuals.^5^

However, it is uncertain whether variants at the 9p21 locus also affect risk of recurrent or subsequent events, including mortality in those with established CHD. Elucidation of this hypothesis would help to better understand its mechanism and estimate its incremental value for stratification of residual risk. Prior studies have shown conflicting results, although most have been underpowered. A literature-based meta-analysis indicated a null association of chromosome 9p21 variants with subsequent CHD events but was based on summary, not individual level data, with varying outcome definitions.^6,7^

The new collaborative GENIUS-CHD (Genetics of Subsequent Coronary Heart Disease) consortium, described in this issue of the journal, was established to investigate genetic determinants of disease progression following an index CHD event.^8^

In this article, we use the GENIUS-CHD resource to: (1) examine the association of variants at the 9p21 locus on risk of subsequent CHD events in individuals with established CHD; (2) compare these to the association between chromosome 9p21 and any CHD observed in the CARDIoGRAMPlusC4D consortium; and (3) explore the potential impact on these estimates of biases that might affect genetic association studies of disease outcome and prognosis.

## Methods

In accordance with Transparency and Openness Promotion Guidelines, the data, analytic methods, and study materials will be made available to other researchers for purposes of reproducing the results or replicating the procedure. Participating studies received local institutional review board approval and included patients who had provided informed consent at the time of enrollment. The central analysis sites also received waivers from their local institutional review board for collating and analysing summary level data from these individual studies. Details about the GENIUS-CHD consortium and study inclusion criteria have been published separately in this issue of the journal,^8^ whereas for this study full details about data sources, genetic variant selection, outcomes and statistical analyses are available in the Data Supplement.

## Results

In total, 49 studies from the GENIUS-CHD consortium contributed to the federated analysis resulting in a sample size of 103 357 individuals of European descent with established CHD and available genotype data at the 9p21 locus. Of these, 93 115 individuals had available data for the primary composite outcome of subsequent CHD death/myocardial infarction (MI), of whom 13 040 experienced these events. Contributing study details are provided in Table. Participant characteristics are representative for populations with established CHD with a weighted mean age of 62.9 years; 73.1% male. As expected, risk factor prevalence was high in this population, including diabetes mellitus (24.4%), hypertension (59.1%), and current smoking (25.7%). Statin use at enrollment varied by study, ranging from 5.2% to 97.3%, with a median of 61.5% (Table).

**Table.**
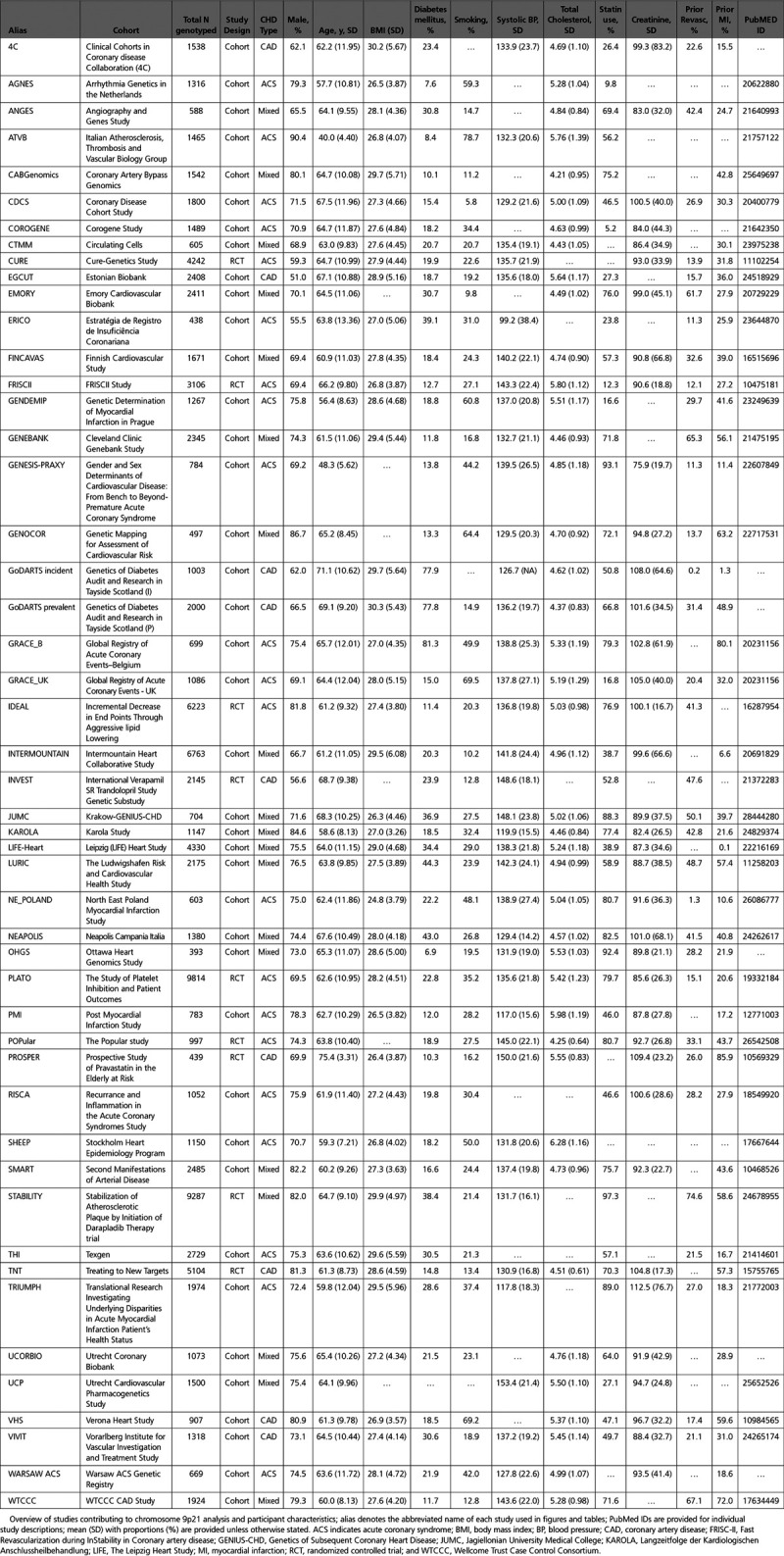
Overview of Studies Contributing to Chromosome 9p21 Analysis and Participant Characteristics

The rs1333049 single nucleotide polymorphism was genotyped in 42 studies, with the remaining 7 studies using highly correlated proxies (*R*^2^>0.90); rs10757278 (4 studies) or rs4977574 (3 studies) when the primary single nucleotide polymorphism was unavailable. Genotyping details are provided in Table I in the Data Supplement. For rs1333049, the average risk allele frequency across the participating studies was 0.518 ranging from 0.453 to 0.587 (Figure I in the Data Supplement).

From CARDIOGRAMplusC4D, after excluding 6 cohorts which had contributed data to both consortia, data were available for association with chromosome 9p21 from 41 studies, including 47 222 cases with CHD and 122 264 controls free of any CHD.

Power to detect different effect sizes, including the effect size identified in CARDIoGRAMplusC4D, using a 2-sided alpha of 0.05, are provided in Table II in the Data Supplement.

### Chromosome 9p21 Association With Subsequent CHD Events

Study-specific results for the association between chromosome 9p21 and risk of the primary outcome of CHD death or MI among individuals with established CHD at baseline, adjusted for age and sex are presented in Figure II in the Data Supplement.

The per-allele odds ratio (OR) for the primary outcome during follow-up was 1.02 (95% CI, 0.99–1.05). The effect estimate again for the primary outcome, based on a time to event analysis and using a Cox regression model, was also similar with a hazard ratio of 1.02 (95% CI, 0.99–1.04; Figure III in the Data Supplement).

**Figure 1.**
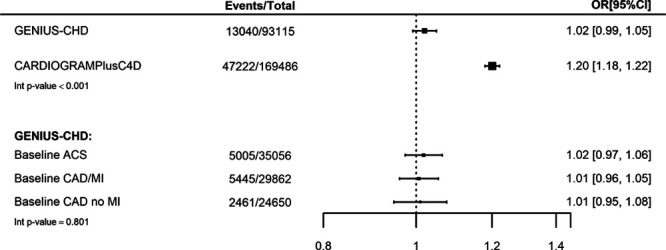
**Association between chromosome 9p21 and subsequent coronary heart disease (CHD) events in all participants with baseline CHD (GENIUS-CHD [Genetics of Subsequent Coronary Heart Disease]) compared with association in CHD cases and CHD-free controls (CARDIoGRAMPlusC4D).** For the CARDIoGRAMPlusC4D consortium (Coronary Artery Disease Genome wide Replication and Meta-analysis [CARDIoGRAM] plus The Coronary Artery Disease [C4D] Genetics) meta-analysis estimate, 6 studies (LURIC, LIFE-Heart [The Leipzig Heart Study], GoDARTS [Genetics of Diabetes Audit and Research in Tayside Scotland], OHGS [Ottawa Heart Genomics Study], PROSPER [Prospective Study of Pravastatin in the Elderly at Risk], WTCCC [Welcome Trust Case Control Consortium]) were excluded as they were also included in GENIUS-CHD. Estimates for GENIUS-CHD are also presented by subtype of CHD at baseline, including acute coronary syndrome (ACS), stable coronary artery disease (CAD) without prior myocardial infarction (MI; CAD/no MI), and stable CAD with prior MI (CAD/MI). All estimates were adjusted for age and sex.

In contrast, a meta-analysis of CARDIOGRAMIplusC4D data (excluding studies also contributing data to GENIUS-CHD), revealed a per-allele OR for a CHD event similar to that reported previously (OR, 1.20; 95% CI, 1.18–1.22). There was evidence of statistical heterogeneity between the estimates (interaction *P*<0.001), Figure 1.

### Subgroup Analyses

We found minimal evidence for heterogeneity in effect estimates when stratifying by CHD subtype at baseline (interaction *P* value 0.801), with no clear evidence for an effect of chromosome 9p21 genetic variation on subsequent CHD death or MI in individuals enrolled with acute coronary syndrome (OR, 1.02; 95% CI, 0.97–1.06), those with coronary artery disease with a prior MI (OR, 1.01; 95% CI, 0.96–1.05), and those with coronary artery disease without prior MI (OR, 1.01; 95% CI, 0.95–1.08, Figure 1).

We further examined the effect of chromosome 9p21 on the primary outcome in prespecified subgroup analyses. We noted a borderline nominally significant interaction with sex, suggesting a greater risk among women with the chromosome 9p21 risk allele, for subsequent CHD death/MI (interaction *P* value = 0.04), whereas nonsignificant trends were noted for greater risk in those without hypertension (*P* value=0.08) or without renal impairment (*P* value=0.17). There were minimal differences in effect estimates by other patient level characteristics including age and diabetes mellitus or according to statin or antiplatelet use or left ventricular impairment at baseline (Figure IV in the Data Supplement).

Similarly, when stratified by study level features, we observed minimal evidence for heterogeneity in effect estimates by study size, geographic region, study design, or length of follow-up (Figure V in the Data Supplement). However, when ordered by date of first enrollment, there was no evidence for variation in effect by time of enrollment (Figure II in the Data Supplement).

### Secondary Outcomes

**Figure 2.**
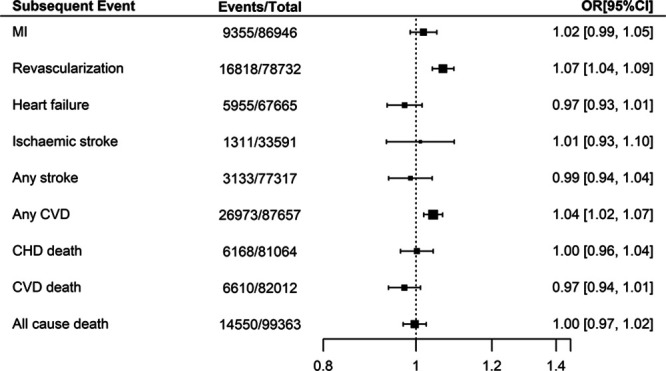
**Association between chromosome 9p21 and secondary outcomes in participants with baseline CHD, within GENIUS-CHD (Genetics of Subsequent Coronary Heart Disease).** All meta-analysis estimates were adjusted for age and sex. CHD indicates coronary heart disease; CVD, cardiovascular disease; MI, myocardial infarction; and OR, odds ratio.

We additionally examined the association between chromosome 9p21 and other subsequent events available for this analysis within the GENIUS-CHD Consortium, listed in Table III in the Data Supplement, with summary estimates provided in Figure 2. Of note, the per-allele effect of risk variants at chromosome 9p21 on subsequent revascularization during follow-up was 1.07 (95% CI, 1.04–1.09). The effect on the composite outcome of any cardiovascular disease, which includes revascularization, was also significant at 1.04 (95% CI, 1.02–1.07). However, there was no clear evidence of association for the remaining secondary outcomes, with only a marginal trend to protection for both subsequent heart failure (OR, 0.97; 95%, CI 0.93–1.01) and cardiovascular disease death (OR, 0.97; 95% CI, 0.94–1.01), as shown in Figure 2.

### Selection Bias

To explore the potential for index event bias, we looked for differences in associations between chromosome 9p21 and known cardiovascular risk factors in the United Kingdom Biobank, among the subset of participants with established CHD, compared with the full UKB cohort (Table IV in the Data Supplement). Although there were differences between the groups in the prevalence or values of the tested risk factors, we did not find clear evidence to indicate a distortion in associations between chromosome 9p21 and age, blood pressure, diabetes mellitus, or smoking. There was, however, a small difference for body mass index, with a greater statistical association between the chromosome 9p21 risk allele and lower body mass index identified in those with established CHD than in the general population (nominal interaction *P* value 0.02, Table IV in the Data Supplement).

We also observed that the chromosome 9p21 risk allele frequency in those surviving with CHD, both in UKB (0.529) and in GENIUS-CHD (0.518, Figure I in the Data Supplement), was higher than the general population in the UKB (0.481) and European reference populations from the 1000 Genomes (Phase 3),^9^ (0.472). This difference in frequency confirms the association of chromosome 9p21 with CHD and also indicated absence of a crude survival bias with loss of large numbers of risk allele carriers to fatal events before entry into CHD cohorts. We did, however, observe a trend to an age association in those with established CHD, as well as the general population in the UKB, with lower chromosome 9p21 risk allele frequencies with advancing age, relative to younger carriers (Figure VI in the Data Supplement).

## Discussion

In this study, we examined the effect of genetic variation at the chromosome 9p21 locus on risk of subsequent events in 103 357 individuals with established CHD using the newly formed GENIUS-CHD consortium.^8^ We found that (1) in contrast to the known strong association with CHD observed in CARDIoGRAMPlusC4D, there was a markedly attenuated and nonsignificant association with subsequent CHD events in GENIUS-CHD; (2) effect estimates in GENIUS-CHD were broadly consistent in stratified analyses based on features related to study design, patient characteristics, and type of index CHD event; and (3) exploratory analyses suggested that selection biases were unlikely to explain the discrepancy. However, we did find evidence of an association between these variants and a secondary outcome of future revascularization events. Our findings, taken together with those from others, support the view that chromosome 9p21 promotes CHD through progressive stable atheroma rather than through development of an unstable phenotype.

The chromosome 9p21 locus is the most widely replicated genetic risk locus for CHD identified to date, with an estimated 15% to 35% increased risk in carriers of the variant allele in prospective population and case-control studies.^5^ However, studies examining the effect on subsequent CHD events in people with known CHD at baseline have reported conflicting results.^10–14^ Our group previously examined this in a literature-based meta-analysis, based on 15 studies with median sample size of 1750 individuals, accruing 25 163 cases of established CHD, and reported no clear evidence of an effect of variants at chromosome 9p21 on the risk of subsequent events.^6^ An analysis by the CHARGE consortium (The Cohorts for Heart and Aging Research in Genomic Epidemiology) of 2953 MI survivors also reported no association with subsequent mortality.^7^ However, the limited size of most prior studies and the limitations of literature meta-analyses indicate that many possible explanations, including errors in risk allele coding and selection biases, could not be adequately explored, precluding meaningful interpretations for any mechanistic or clinical implications.

The emergence of the GENIUS-CHD Consortium has now permitted a robust evaluation of the role of chromosome 9p21 in subsequent CHD event risk, revealing a clear lack of association with a common composite coronary end point. This is in marked contrast to findings from studies comparing cases to CHD-free controls, as confirmed through meta-analysis of CARDIoGRAMPlusC4D data. Furthermore, we were able to add to previous findings by showing that the type of CHD at baseline, whether acute coronary syndrome or stable CHD with or without prior MI, does not alter this association. We also interrogated several widely proposed explanations that could account for our findings through prespecified subgroup analyses and confirmed that most of these, specifically older age, medication use at baseline (statin or antiplatelet), study size or follow-up duration, did not appreciably alter the association findings. Our finding of a possible interaction with sex, warrants further investigation but should be considered hypothesis-generating given the borderline evidence of an interaction.

Selection bias (ie, index event bias or collider-stratification bias) could potentially explain reversed or attenuated associations in disease progression studies like this, operating by inducing relationships between (otherwise independent) risk factors through the selection of individuals with disease.^15,16^ Specifically, individuals surviving a first event consequent on exposure to a particularly strong risk factor may have lower levels of exposure to other individually weaker, independent risk factors, which can then attenuate the association of the risk factor of interest with subsequent events. However, the distribution of common risk factors by chromosome 9p21 genotype did not differ when compared between the general population and the subset with CHD in the UKB, using interaction tests. The only exception was for body mass index, a potentially differential association with chromosome 9p21 in those with CHD compared with the general population was noted. However, the effect size was small in both populations and on its own is unlikely to indicate presence of substantial index event bias.

Selection bias may also theoretically occur by focusing on subjects surviving a first event, where chromosome 9p21 risk allele carriers at risk of fatal CHD events are lost before enrollment into CHD cohorts, thereby diluting the future impact of the variant on subsequent CHD events. In this scenario, we would expect a lower risk allele frequency in those surviving CHD and entering CHD cohorts, but we found no evidence for this. Among those with CHD in the UKB, and among the whole UKB cohort, we did find a progressive loss of risk allele carriers with increasing age, consistent with prior findings of a greater association with CHD, among younger individuals in case-control studies.^5^ Given patients with CHD are generally older, it is possible that a subtle survival bias may still be influencing our findings, although all analyses were adjusted for age. However, based on simulation modeling, sample size, and projected single nucleotide polymorphism effect size, we and others have previously estimated that selection biases are only minimally operating in this context and would be unlikely to account for our observed findings.^17,18^ Although our findings potentially argue against important selection biases in the analysis for the primary outcome, they are relatively insensitive assessments and may not fully elucidate such biases.

Possible biological explanations could also exist for our findings. Pathological studies indicate differences between chronic stable atherosclerotic plaques that cause ischemia through progressive vessel occlusion and vulnerable plaques with thin caps, prone to sudden plaque rupture, unheralded MI, and coronary deaths.^19^ In a seminal study dissecting the phenotype of CHD, a lack of effect for chromosome 9p21 and MI was noted, when both cases and controls had underlying atherosclerosis.^20^ Our group and others have in parallel shown that chromosome 9p21 robustly associates with atherosclerotic phenotypes,^21^ whereas functional studies have also implicated this region with molecular activity that drives atheroma.^22^ Furthermore, in this study, we show that the only outcome positively associated with chromosome 9p21 is incident revascularization, perhaps reflecting more severe atherosclerosis burden. Collectively, these data support the concept that chromosome 9p21 promotes progressive atheroma formation and does not confer risk via plaque rupture.

In this context, it is worth noting that chromosome 9p21 associates more robustly with CHD in case-control studies than in prospective cohort studies.^7^ The difference, as proposed by others, could hypothetically be accounted for by incidence-prevalence bias, with chromosome 9p21 carriers more likely to survive a CHD event and thus be over represented among CHD cases (the opposite to survival bias described above).^7^ This becomes more likely as stated above if chromosome 9p21 drives a more progressive and stable atheroma phenotype. If this holds true, then among survivors with established CHD, one might expect that chromosome 9p21 carriers could hold a small favorable advantage over those who experience CHD in its absence, due instead to other more dangerous or vulnerable characteristics, and despite undergoing more subsequent revascularization, these chromosome 9p21 carriers do not experience more dangerous or fatal events.

These findings have important implications. Clinically, they indicate that a degree of caution should be applied when considering or evaluating patients for chromosome 9p21 to predict disease progression or residual risk. They also highlight the need to appreciate important biases that may inflate or attenuate association findings in the setting of subsequent events for individuals with established disease. Mechanistically, these findings support existing and emerging efforts seeking to elucidate the mechanism of the most robust genetic discovery for CHD in recent decades.

There are important limitations to consider. First, among individuals in GENIUS with established CHD, the timing of the first CHD event or age of onset was often unknown, so we could not account for this variable in our analyses. However, the lack of association in the acute coronary syndrome studies, which had documented timing of the first event, suggests this did not impact the findings. Second, we had limited information on whether subsequent revascularization events were late staged procedures, which would count as part of the index CHD event or unplanned and symptom driven and thereby a true subsequent event, which may have diluted the effect estimate. Third, although we did not observe a specific interaction for statin or aspirin use, we cannot rule out an effect of combined or additional medication usage attenuating the association signal, given the high prevalence of secondary prevention drug use in this setting compared with general population cohorts. Fourth, our analyses were restricted to participants of European descent as most of the included studies only recruited these individuals, and so we were markedly underpowered to explore associations in other ethnic groups. Unfortunately, this remains a wider problem of genetic research and global efforts are ongoing to address this imbalance. Finally, variability of follow-up duration across studies is an analytical challenge and could have impacted our findings, through misclassification. However, a sensitivity analysis stratifying on the follow-up duration of individual studies (<5 or 5≥ years) revealed minimal evidence (*P*=0.62) of heterogeneity in effect estimates (Figure V in the Data Supplement), suggesting that this is unlikely to have influenced our findings significantly as effect estimates were concordant across studies with different lengths of follow-up. Our major strengths, however, include the size of the study and the large number and types of subsequent events and an effort to examine for selection biases. We also sought to mitigate potential miscoding of the risk allele, given rs1333049 is a palindromic single nucleotide polymorphism, and also the risk allele C changes from being a minor allele in population cohorts to the major allele in CHD cohorts. Finally, this analysis benefitted from the collective expertise and input of over 170 investigators and analysts, many of whom have previously reported on chromosome 9p21.

In conclusion, using the newly formed GENIUS-CHD consortium, we demonstrate that variation at chromosome 9p21 shows no clear association with risk of subsequent CHD events when all individuals have established CHD at baseline. This is in marked contrast to prior case-control studies examining odds of CHD presence compared with disease-free controls. We could not account for the attenuation of effect in terms of selection biases or subgroup effects. However, we did find a greater risk for incident revascularization in those with established CHD, and although residual bias may be at play, our findings collectively support the view that chromosome 9p21 promotes CHD through progressive stable atheroma rather than through development of an unstable phenotype.

## Acknowledgments

The GENIUS-CHD (Genetics of Subsequent Coronary Heart Disease) collaborators would like to express their immense gratitude to all patients who participated in each of the individual studies as well as the many personnel who helped with recruitment, collection, curation, management and processing of the samples and data. We also thank the CARDIoGRAMPlusC4D steering committee for providing the summary data after excluding the cohorts already in GENIUS-CHD.

## Sources of Funding

The funder(s) of the study had no role in study design, data collection, data analysis, data interpretation, or writing of the report. Within GENIUS-CHD (Genetics of Subsequent Coronary Heart Disease), all participating investigators and sponsors who contributed data and analyses are acknowledged irrespective of academic or industry affiliations. Specific funding statements: Dr Patel is funded by a British Heart Foundation Intermediate Fellowship (FS/14/76/30933). This research was also supported by the National Institute for Health Research University College London Hospitals Biomedical Research Centre; Dr Schmidt is funded by BHF grant PG/18/5033837; Dr Holmes works in a unit that receives funding from the UK Medical Research Council and is supported by a British Heart Foundation Intermediate Clinical Research Fellowship (FS/18/23/33512) and the National Institute for Health Research Oxford Biomedical Research Centre; The AGNES study (Arrhythmia Genetics in the Netherlands) was supported by research grants from the Netherlands Heart Foundation (2001D019, 2003T302, 2007B202 and the PREDICT project (CVON 2012-10)), the Leducq Foundation (grant 05-CVD) and the Center for Translational Molecular Medicine (CTMM COHFAR); The Cleveland Clinic Genebank Study was supported in part by NIH (National Institutes of Health) grants R0133169, R01ES021801, R01MD010358, and R01ES025786, R01HL103866, R01DK106000, R01HL126827, P20HL113452, P01HL098055, P01HL076491, and R01HL103931; The 4C study (Clinical Cohorts in Coronary disease Collaboration) was supported in part by NIHR and Barts Charity; The Corogene study was supported by grants from Aarno Koskelo Foundation, Helsinki University Central Hospital special government funds (EVO no. TYH7215, no. TKK2012005, no. TYH2012209, no. TYH2014312), and Finnish Foundation for Cardiovascular research; CABGenomics was supported by Stanton Shernan, C. David Collard, Amanda A. Fox/R01 HL 098601 National Heart Long and Blood Institute; The CDCS (Coronary Disease Cohort Study) and PMI (Post Myocardial Infarction Study) were funded by the Health Research Council and Heart Foundation of New Zealand; Dr Samman-Tahnan is supported by the National Institutes of Health/ National Institutes of Aging grant AG051633; Dr Sandesara is supported by the Abraham J. & Phyllis Katz Foundation (Atlanta, GA); The Emory Cardiovascular Biobank is supported by NIH grants 5P01HL101398-02, 1P20HL113451-01, 1R56HL126558-01, 1RF1AG051633-01, R01 NS064162-01, R01 HL89650-01, HL095479-01, 1U10HL110302-01, 1DP3DK094346-01, 2P01HL086773-06A1; this Estonian Biobank was funded by EU H2020 grant 692145, Estonian Research Council Grant IUT20-60, IUT24-6, PUT1660, PUT735 and European Union through the European Regional Development Fund Project No.2014-2020.4.01.15-0012 GENTRANSMED, NIH-GIANT, ERA-CVD grant Detectin-Heart failure and 2R01DK075787-06A1; GENESIS-PRAXY (Gender and Sex Determinants of Cardiovascular Disease: From Bench to Beyond-Premature Acute Coronary Syndrome) is funded by the Canadian Institutes of Health Research and Heart and Stroke Foundations of Alberta, NWT & Nunavut, British Columbia and Yukon, Nova Scotia, Ontario, and Quebec (HSFC); The GENDEMIP study (Genetic Determination of Myocardial Infarction in Prague) was supported by Project (MH, Czech Republic) No. 00023001 (Institute of Clinical and Experimental Medicine, Prague); GoDARTS (Genetics of Diabetes Audit and Research in Tayside Scotland) was funded by the Wellcome Trust (072960/Z/03/Z, 084726/Z/08/Z, 084727/Z/08/Z, 085475/Z/08/Z, 085475/B/08/Z) and as part of the EU IMI-SUMMIT programme. C.N.P. has received grant funding from the Wellcome Trust to develop the GoDARTS cohort; Dr Mordi is supported by an NHS Education of Scotland/Chief Scientist Office Postdoctoral Clinical Lectureship (PCL 17/07); the GENECOR study (Genetic Mapping for Assessment of Cardiovascular Risk) was supported in part by the Italian Ministry of Research’s Fund for Basic Research (FIRB 2005); GRACE (Global Registry of Acute Coronary Events–Belgium) UK was supported in part by an Educational Grant from Sanofi Aventis; Award from Chief Scientist Office, Scotland; INVEST-GENES (International Verapamil SR Trandolopril Study Genetic Substudy) was supported by the National Institute of Health Pharmacogenomics Research Network grant U01-GM074492, NIH R01 HL074730, University of Florida Opportunity Fund, BASF Pharma and Abbott Laboratories; Italian Atherosclerosis, Thrombosis and Vascular Biology Group was supported by Epidemiologia e Genetica della Morte Improvvisa in Sardegna; The KAROLA study has received financial support by the German Ministry of Education and Research (01GD9820/0 and 01ER0814), by the Willy-Robert-Pitzer Foundation, and by the Waldburg-Zeil Clinics Isny; The KRAKOW GENIUS Study was supported by a grant from the Polish Ministry of Science and Higher Education, no. NN402083939 and the National Science Centre, no. 2013/09/B/NZ5/00770; LIFE-Heart was funded by the Leipzig Research Center for Civilization Diseases (LIFE). LIFE is an organizational unit affiliated to the Medical Faculty of the University of Leipzig. LIFE is funded by means of the European Union, by the European Regional Development Fund (ERDF) and by funds of the Free State of Saxony within the framework of the excellence initiative; The LURIC study (The Ludwigshafen Risk and Cardiovascular Health Study) was supported by the Seventh Framework Program (AtheroRemo, grant agreement number 201668 and RiskyCAD (Personalized Diagnostics and Treatment of High Risk Coronary Artery Disease Patients), grant agreement number 305739) of the European Union; The NEAPOLIS CAMPANIA (Neapolis Campania Italia) study was suppported by European Research Council Advanced Grant (CardioEpigen, no. 294609);Italian Ministry of Health (PE-2013-02356818);Italian Ministry of Education, University and Research (2015583WMX); The North East Poland Myocardial Infarction Study was supported by grant N N 402 529139 from the National Science Center (Poland); Dr Vilmundarson is supported by a graduate fellowship of the University of Ottawa Heart Institute; OHGS (Ottawa Heart Genomics Study) was funded in part by a Heart and Stroke Foundation grant; Dr Stott was supported in part by an investigator initiated grant from Bristol Myers Squibb USA; The PROSPER study (Prospective Study of Pravastatin in the Elderly at Risk) was supported by an investigator initiated grant obtained from Bristol-Myers Squibb. Dr Jukema is an Established Clinical Investigator of the Netherlands Heart Foundation (grant 2001 D 032). Support for genotyping was provided by the seventh framework program of the European commission (grant 223004) and by the Netherlands Genomics Initiative (Netherlands Consortium for Healthy Aging grant 050-060-810); The RISCA (Recurrance and Inflammation in the Acute Coronary Syndromes Study) was supported in part by FRSQ, HSFC, Merck Frost Canada, Pfizer Canada; The SHEEP study (Stockholm Heart Epidemiology Program) was supported by grants from the Swedish Council for Work Life and Social Research, and the Stockholm County Council; The TNT trial (Treating to New Targets) was sponsored by Pfizer who granted access to data, Genotyping of the samples was funded in part by grants from Genome Canada and Genome Quebec and the Canadian Institutes of Health Research (CIHR); Dr Arsenault holds a junior scholar award from the Fonds de recherche du Quebec- Sante (FRQS); Dr Cresci is supported, in part, by the National Institutes of Health (Cresci R01 NR013396). The TRIUMPH study (Translational Research Investigating Underlying Disparities in Acute Myocardial Infarction Patient’s Health Status) was sponsored by the National Institutes of Health: Washington University School of Medicine SCCOR Grant P50 HL077113; The Utrecht Cardiovascular Pharmacogenetics Study studies were funded by the Netherlands Heart Foundation and the Dutch Top Institute Pharma Mondriaan Project; The Verona Heart Study was supported by the Cariverona Foundation; Veneto Region; Italian Ministry of Education, University, and Research (MIUR); LURM (Laboratorio Universitario di Ricerca Medica) Research Center, University of Verona; The Warsaw ACS Registry (acute coronary syndrome) is supported by grant N R13 0001 06 from The National Centre for Research and Development (NCBiR), Statutory Grant from Medical University of Warsaw; Dr Nelson is funded by the British Heart Foundation; Prof. Samani is funded by the British Heart Foundation and is a NIHR Senior Investigator. Prof Hingorani is a NIHR Senior Investigator; Prof Asselbergs is supported by University College London Hospitals NIHR Biomedical Research Centre, EU/EFPIA Innovative Medicines Initiative 2 Joint Undertaking BigData@Heart grant n° 116074, the European Union’s Horizon 2020 research and innovation programme under the ERA-NET Co-fund action N°01KL1802 (Druggable-MI-gene) jointly funded by the Dutch Heart Foundation and Netherlands Organization for Health Research and Development (ZonMw).

## Disclosures

Dr Patel has received speaker fees and honoraria from Amgen, Sanofi and Bayer and research grant funding from Regeneron; Dr Holmes has collaborated with Boehringer Ingelheim in research, and in accordance with the policy of The Clinical Trial Service Unit and Epidemiological Studies Unit (University of Oxford), did not accept any personal payment; Dr Akerblom has received institutional research grant and speakers fee from AstraZeneca, institutional research grant from Roche Diagnostics; Dr James has received grants from AstraZeneca, The Medicines Company, Swedish heart and lung foundation, Swedish research council, Janssen; personal fees from Bayer; Dr Hagstrom declares being an expert committee member, lecture fees, and institutional research grant from Sanofi, and Amgen; institutional research grants from AstraZeneca, and GlaxoSmithKline; expert committee member and lecture fees NovoNordisk and Behringer; Dr Held declares institutional research grant, advisory board member and speaker’s bureau from AstraZeneca; institutional research grants from Bristol-Myers Squibb Merck & Co, GlaxoSmithKline, Roche Diagnostics. Advisory board for Bayer and Boehringer Ingelheim; Dr Lindholm has received institutional research grants from AstraZeneca, and GlaxoSmithKline; Speaker fees from AstraZeneca, Speaker fees from AstraZeneca; Dr Siegbahn has received institutional research grants from AstraZeneca, Boehringer Ingelheim, Bristol-Myers Squibb/Pfizer, Roche Diagnostics, GlaxoSmithKline; Dr ten Berg reports receiving fees for board membership from AstraZeneca, consulting fees from AstraZeneca, Eli Lilly, and Merck, and lecture fees from Daiichi Sankyo and Eli Lilly, AstraZeneca, Sanofi and Accumetrics; Dr Wallentin reports institutional research grants, consultancy fees, lecture fees, and travel support from Bristol-Myers Squibb/Pfizer, AstraZeneca, GlaxoSmithKline, Boehringer Ingelheim; institutional research grants from Merck & Co, Roche Diagnostics; consultancy fees from Abbott; and holds a patent EP2047275B1 licensed to Roche Diagnostics, and a patent US8951742B2 licensed to Roche Diagnostics; Dr Claes reports lecture fees, and an institutional research grant from Sanofi, and Amgen; institutional research grants from AstraZeneca, and GlaxoSmithKline; and lecture fees from NovoNordisk and AstraZeneca. Dr Asselbergs has received research funding from Regeneron, Pfizer, Sanofi. The other authors report no conflicts.

## Supplementary Material
