# Supplementary material for: Association of Chromosome 9p21 With Subsequent Coronary Heart Disease Events: A GENIUS-CHD Study of Individual Participant Data
Source: Circ Genom Precis Med. 2019 Apr 16;12(4):e002471. doi: 10.1161/CIRCGEN.119.002471 (PMC6625876; doi:10.1161/CIRCGEN.119.002471)
Supplement: Supplementary file 1 [file hcg-12-e002471-s001.pdf]

SUPPLEMENTAL MATERIAL

1 Supplemental Methods

Data sources

The GENIUS-CHD consortium is an international collaboration of prospective cohort studies selectively including individuals with established coronary heart disease at baseline and following them for future subsequent CHD events, with cases defined as those experiencing a subsequent event and controls as those who do not. [1 2] Full details on the individual studies have been published previously, with study-specific references provided in Table 1 (main paper).

The primary criteria for inclusion in the consortium are studies that recruited individuals with: (1) established CHD, defined as a history of or presence at baseline of acute coronary syndrome, or of coronary artery disease as evidenced by any revascularization procedure such as percutaneous coronary intervention or coronary bypass surgery, or a significant (50%) coronary artery plaque at angiography affecting any major epicardial vessel, (2) availability of prospective follow-up and ascertainment of at least one clinical cardiovascular outcome including all-cause mortality, and (3) availability of samples and/or biomarkers or in-silico genotyping data. Full details about the GENIUS-CHD Consortium have been published elsewhere.[1]

The CARDIOGRAMPlusC4D Consortium is a global collaboration that, in contrast to GENIUS-CHD, includes only case-control studies, where cases are defined as having any CHD and controls are individuals free of CHD. The publicly-available dataset consists of individuals predominantly of European descent, with imputed genome-wide data covering 6.7million common variants and 2.7million low-frequency variants. Full details of this resource have been published elsewhere. [3]

The UK Biobank recruited 500,000 participants aged 40-69 from England, Scotland and Wales between 2006 and 2010, with comprehensive baseline data and prospective follow up recorded. We used these data for sensitivity analyses to look for any evidence of selection bias. At the time of our

analyses genetic data were available for 408,480 participants. Full details of this resource have been published elsewhere. [4]

Genetic variant selection

We selected the genetic variant rs1333049 as the most established marker for CHD risk at the 9p21 locus. Genotype data were subjected to local quality controls by each study prior to analysis. Given the palindromic nature of rs1333049 (G/C), and the potential for erroneous coding, all studies were asked to confirm the SNP was reported on the forward (upper/+) DNA strand. Studies were also asked to run association analysis according to the established risk allele (C), not the minor allele given the risk allele frequency fluctuates around 0.5. At the meta-analysis level, these risk allele frequencies were again examined to ensure that they were broadly representative across studies, see Supplementary Figure 1. Genotyping details (including SNP and linkage disequilibrium with the lead variant) for each cohort in GENIUS-CHD are provided in Supplementary Table 1.

Outcomes

The primary outcome of interest was a composite of CHD death or myocardial infarction, whichever came first (CHD death/MI) during follow-up. Secondary outcomes of interest during follow-up included: MI, coronary revascularization, heart failure, ischemic stroke, any stroke, any cardiovascular disease (CVD, including MI, stroke, revascularization, and CVD death), CHD death, CVD death, and all-cause death.

Statistical analysis

Individual studies participating within the GENIUS-CHD Consortium evaluated the association between chromosome 9p21 variants and subsequent events assuming an additive genetic model and using both binary logistic regression and time-to-event Cox proportional-hazards models. All analyses were adjusted for age and sex. Across the consortium, analyses were performed using

shared statistical scripts and harmonized datasets, as described in the accompanying design paper.[1] For the purposes of comparison with CARDIOGRAMplusC4D, which reported odds ratios (OR), we present logistic regression results as our primary analysis, although time-to-event results are also provided and can be found in the supplementary materials.

For both GENIUS-CHD and CARDIOGRAMplusC4D, study-level effect estimates and their corresponding standard errors were then meta-analysed using an inverse variance weighted fixed-effects model. Heterogeneity was quantified using the χ^2 test for heterogeneity and the I^2 statistic.

To assess consistency, the chromosome 9p21 association with the primary outcome was stratified on patient-level characteristics measured at baseline, including: age (< or ≥ 65years), sex, hypertension (physician diagnosed or treated), type 2 diabetes (T2DM, physician diagnosed or treated), body mass index (BMI 18.5-24.9; 25-29.9; ≥30kg/m^2), statin use, anti-platelet use, renal impairment (eGFR <60ml/kg/min), and left-ventricular impairment (LVEF<45% or diagnosed heart failure with impaired systolic function). The meta-analysis was also stratified on study-specific factors including: sample size (< or ≥ 1000 participants), geographical location (by continent), study design, and follow-up years (< or ≥ 5 years). Differences between estimates were evaluated using interaction tests.[5, 6] Finally, to explore the impact of time period of recruitment, stratification by enrollment date was not possible due to the variable duration, and dynamic ongoing enrollment in many participating studies. As such, study specific results were ordered by enrollment start date to assess for crude differences over time.

To assess the potential for index event bias in our data,[7] (whereby associations between the exposure of interest and risk factors for the disease may be induced by conditioning on subjects with CHD), we examined for any differences in associations between chromosome 9p21 and common cardiovascular risk factors in individuals from the general population and those with established CHD. For this purpose we utilized the UK Biobank (UKB) which has available genetic and risk factor data for both groups of individuals. Risk factors tested included age, smoking behaviour, T2DM, BMI, and systolic blood pressure (SBP). Biomarkers were unavailable at the time of analysis. Differences between risk

factor associations in each group (general population and those with CHD) were tested using interaction effects. We also explored potential for survival bias by looking for differences in chromosome 9p21 risk allele frequencies in survivors with CHD compared to the general population in the UKB cohort and then by 5-year age categories in each group.

To reduce the potential for population stratification bias, we excluded individuals that self-identified as non-European. Effect sizes and confidence intervals (CI) were calculated using a two-sided alpha of 0.05, and results are presented as mean difference, ORs, or hazard ratios (HR). All analyses were conducted using R software (R Development Core Team).[8]

2. Supplemental Tables

Table 1: Genotyping details for each cohort

Alias	SNP used	R^2 with lead SNP	Risk allele	Risk allele frequency	HWE	Genotyping Platform
4C	rs1333049		C	0.505	0.919	Custom
AGNES	rs4977574	0.878	G	0.485	0.136	Illumina Human610k-Quad v1 array
ANGES	rs1333049		C	0.484	0.871	Metabochip
ATVB	rs1333049		C	0.585	1.000	Affymetrix 6.0 GeneChip
CABGenomics	rs1333049		C	0.560	0.757	Sequenom
CDCS	rs1333049		C	0.513	0.451	Sequenom and Taqman assays
COROGENE	rs1333049		C	0.466	0.603	Illumina Human610k-Quad
CTMM	rs1333049		C	0.537	0.326	Affymetrix Axiom tx
CURE	rs10757278	0.968	G	0.527	0.176	Taqman assay
EGCUT	rs1333049		C	0.479	0.094	Illumina OmniExpress, Illumina Global Screening array
EMORY	rs1333049		C	0.503	0.515	Multiplex or taqman
ERICO	rs1333049		C	0.487	1.000	Affymetrix axion
FINCAVAS	rs1333049		C	0.453	0.657	Metabochip, n = 2278, CoreExome, n = 926
FRISCII	rs1333049		C	0.485	0.747	
GENDEMIP	rs1333049		C	0.526	0.574	Taqman assay
GENEBANK	rs1333049		C	0.544	0.647	Affymetrix 6.0 GeneChip
GENESIS-PRAXY	rs4977574	0.878	G	0.555	1.000	Sequenom: Platform: iPLEX© Gold Genotyping Technology Taqman: Platform: TaqMan© Genotyping Technology (Applied Biosystems)
GENOCOR	rs1333049		C	0.560	1.000	High resolution melting curve analysis-LC Green and a Light Scanner (Idaho Tech)
GoDARTSincident	rs1333049		C	0.511	0.231	Affy; Affy1KG; Illumina; Illumina 1KG; broad
GoDARTSprevalent	rs1333049		C	0.507	0.227	Affy; Affy1KG; Illumina; Illumina 1KG; broad
GRACE	rs1333049		C	0.516	0.150	Sequenom (734 samples)
GRACE_UK	rs1333049		C	0.518	0.466	Sequenom

IDEAL	rs1333049		C	0.501	0.704	Sequenom MassArray Maldi-TOF System
INTERMOUNTAIN	rs1333049		C	0.520	0.144	Applied Biosciences Inc
INVEST	rs10757278	0.968	G	0.498	0.931	Taqman; Illumina IBC; Illumina OmniExpress
JUMC	rs1333049		C	0.509	0.707	TaqMan? Real-Time PCR Assays (7900HT Fast Real Time System or OpenArray NT cycler)
KAROLA	rs1333049		C	0.524	0.813	restriction fragment length polymorphism (RFLP)
LIFE-Heart	rs1333049		C	0.506	0.855	Affymetrix Axiom CADLIFE (CEU+custom content) or Affymetrix Axiom CEU
LURIC	rs1333049		C	0.533	0.005	Affymetrix 6.0
NE_POLAND	rs1333049		C	0.522	0.087	ABI 7500 real time PCR
NEAPOLIS	rs1333049		C	0.542	0.001	GENOTYPING 7900HT Fast Real-Time PCR System
OHGS	rs1333049		C	0.527	0.129	Affymetrix Axiom
PLATO	rs1333049		C	0.518	0.731	Illumina HumanOmni2.5-4v1 BeadChip, Illumina Infinium HumanOmniExpressExome-8 v1
PMI	rs1333049		C	0.521	0.032	Sequenom and Taqman assays
POPular	rs1333049		C	0.505	0.145	
PROSPER	rs1333049		C	0.556	0.290	Illumina 660K quad beadchip
RISCA	rs4977574	0.878	G	0.563	0.316	iPLEX technology on a MassARRAY Compact Analyser
SHEEP	rs1333049		C	0.482	0.723	Illumina Cardiometabochip
SMART	rs10757278	0.968	G	0.515	0.460	TaqMan assay
STABILITY	rs1333049		C	0.534	0.723	Illumina HumanOmniExpressExome-8 v1 BeadChip
THI	rs1333049		C	0.531	0.673	Sequenom massarray system, TaqMan
TNT	rs1333049		C	0.524	0.844	Sequenom massarray Maldi-tof System
TRIUMPH	rs1333049		C	0.522	0.191	Infinium HumanCore BeadChip array (GWAS) and the Illumina HumanExome v1.1 Analysis BeadChip array (Exome)
UCORBIO	rs1333049		C	0.513	0.541	TaqMan assay
UCP	rs1333049		C	0.501	0.504	50K Illumina CARe iSelect (IBC)
VHS	rs1333049		C	0.586	0.274	iPLEX MassARRAY platform, Multilocus genotyping assay by Roche
VIVIT	rs1333049		C	0.533	0.699	CardioMetaboChip
WARSAW ACS	rs10757278	0.968	G	0.496	0.165	TaqMan Assay; ABI 7500 real time PCR platform
WTCCC	rs1333049		C	0.554	0.712	Affymetrix GeneChip© Human Mapping 500K Array Set

Table 2: Power of the rs1333049 SNP association with CHD death or MI

OR	Power
1.02	0.40
1.03	0.66
1.04	0.86
1.05	0.96
1.10	1.00
1.20	1.00

N.b. based on an alpha of 0.10 (two sided 0.05)

Table 3: Subsequent events available in each study for association with 9p21.

Alias	CHD Death or MI	Myocardial Infarction	Revasc	Ischemic Stroke	Any Stroke	Heart Failure	All CVD	CHD Death	CVD Death	All Cause Death	Mean Follow-up (SD), years
4C	22	NA	NA	NA	NA	NA	63	22	25	63	2.38 (0.75)
AGNES	155	124	232	NA	38	82	334	38	38	180	7.10 (4.74)
ANGES	173	86	207	60	66	124	310	128	89	178	8.20 (4.40)
ATVB	235	229	544	NA	22	NA	338	40	30	81	10.42 (4.48)
CABGenomics	NA	NA	NA	NA	NA	156	NA	NA	NA	393	NA
CDCS	532	522	495	116	146	337	863	196	242	433	5.20 (2.14)
COROGENE	NA	NA	NA	NA	NA	NA	NA	NA	NA	359	NA
CTMM	12	10	70	NA	NA	NA	75	2	NA	4	0.97 (0.36)
CURE	362	257	146	NA	50	106	449	176	78	246	0.78 (0.28)
EGCUT	640	455	230	301	326	1468	1097	284	580	825	6.56 (2.90)
EMORY	386	132	735	NA	65	196	1017	328	359	595	5.83 (3.14)
ERICO	88	63	NA	NA	NA	NA	111	70	70	111	2.88 (1.47)
FINCAVAS	447	309	399	191	214	336	730	247	185	440	8.57 (3.99)
FRISCII	666	510	1679	NA	NA	NA	1914	266	251	362	7.46 (2.09)
GENDEMIP	NA	NA	NA	NA	NA	NA	NA	NA	93	155	1.12 (0.78)
GENEBANK	116	116	857	NA	30	NA	997	NA	NA	153	3.00 (0.00)
GENESIS-PRAXY	36	28	53	NA	1	3	72	NA	29	8	1.00 (0.00)
GENOCOR	35	32	80	NA	12	3	141	24	26	57	5.68 (1.20)
GoDARTSincident	159	128	157	32	70	93	553	85	115	286	3.64 (2.99)
GoDARTSprevalent	416	329	172	82	173	251	1225	260	322	750	6.78 (2.93)
GRACE	47	47	NA	NA	NA	NA	NA	NA	2	NA	4.25 (1.79)
GRACE_UK	269	184	319	NA	54	194	990	164	NA	230	10.62 (2.15)
IDEAL	464	398	948	7	179	136	1307	89	119	203	4.80 (0.41)

INTERMOUNTAIN	2749	1664	2009	NA	494	650	3667	1609	1643	2317	8.56 (5.39)
INVEST	63	63	NA	NA	49	NA	166	NA	21	76	3.08 (0.81)
JUMC	89	75	47	14	14	20	130	28	30	81	0.85 (0.33)
KAROLA	185	135	NA	NA	77	NA	268	104	139	255	11.67 (2.94)
LIFE-Heart	58	NA	NA	NA	NA	NA	62	58	62	391	1.76 (2.19)
LURIC	471	146	NA	NA	42	NA	471	413	457	736	8.65 (3.14)
NE_POLAND	NA	NA	NA	NA	NA	NA	NA	NA	NA	189	7.20 (2.75)
NEAPOLIS	27	24	14	5	5	12	201	13	13	45	1.07 (0.55)
OHGS	24	15	117	NA	NA	NA	137	13	17	18	1.77 (0.29)
PLATO	793	567	2140	89	110	423	2686	300	326	369	0.86 (0.24)
PMI	313	313	338	67	85	157	532	186	221	374	8.66 (3.32)
POPular	66	58	49	12	12	NA	116	8	8	16	1.00 (0.00)
PROSPER	86	86	11	NA	29	32	125	37	46	69	3.14 (0.68)
RISCA	139	128	584	NA	6	30	619	25	27	34	1.22 (0.18)
SHEEP	483	483	5	158	188	284	702	155	178	595	14.87 (5.91)
SMART	257	163	868	NA	58	NA	NA	118	NA	259	7.57 (3.51)
STABILITY	730	450	1038	162	181	212	1582	351	387	621	3.60 (0.57)
THI	309	309	570	NA	86	398	1234	NA	NA	632	5.49 (3.43)
TNT	347	279	825	NA	130	144	1098	97	118	251	4.57 (1.19)
TRIUMPH	NA	NA	NA	NA	NA	NA	NA	NA	NA	97	0.97 (0.15)
UCORBIO	59	59	101	NA	9	24	155	NA	17	36	NA
UCP	229	229	547	27	53	90	NA	NA	NA	NA	8.01 (4.16)
VHS	132	24	NA	NA	NA	NA	NA	109	126	182	5.62 (2.99)
VIVIT	171	126	237	NA	80	NA	436	99	131	290	7.58 (2.78)
WARSAW ACS	NA	NA	NA	NA	NA	NA	NA	NA	NA	106	2.96 (1.17)
WTCCC	NA	NA	NA	NA	NA	NA	NA	NA	NA	411	10.05 (2.81)

CHD = Coronary Heart Disease; CVD = Cardiovascular

Table 4: Risk factor distribution by Ch9p21 genotype in the whole UKB population (n = 408,480) and the subset with CHD (n=15,275).

N	UKB General Population (N=408,480)					UKB CHD Population (N=15,275)					Difference in Association	
rs1333049 genotype	**CC**	**GC**	**GG**	**MD or OR (95%CI)**		**CC**	**GC**	**GG**	**MD or OR (95%CI)**		**OR Interaction**	**P value**
Age, yrs	56.85	56.92	56.97	-0.06 (-0.09, -0.03)		62.01	62.3	62.34	-0.17 (-0.30, -0.04)		0.11 (-0.02, 0.25)	0.105025
Ever Smoked, %	45.04%	45.27%	45.39%	0.99 (0.98, 1.00)		65.30%	67.00%	65.50%	0.99 (0.94, 1.04)		1.00 (0.95,1.05)	0.926777
Type 2 Diabetes, %	5.00%	4.85%	4.68%	1.04 (1.02, 1.06)		17.67%	17.97%	16.86%	1.03 (0.97,1.09)		1.00 (0.95-1.07)	0.81107
Body Mass Index, kg/m2	27.18	27.21	27.21	-0.02 (-0.04, 0.01)		28.83	29.03	29.04	-0.15 (-0.26, -0.04)		0.13 (0.02, 0.24)	0.020743
Systolic Blood Pressure, mmHg	139.9	139.8	139.8	0.06 (-0.03, 0.15)		139.7	140.1	140.1	-0.16 (-0.63, 0.30)		0.23 (-0.25, 0.70)	0.349446

CHD defined as prior MI or revascularization. Mean difference (MD), odds ratio (OR), 95% confidence interval (95%CI), difference were calculated either on the identity scale or on the natural logarithm of the OR.

3 Supplemental Figures

Figure 1

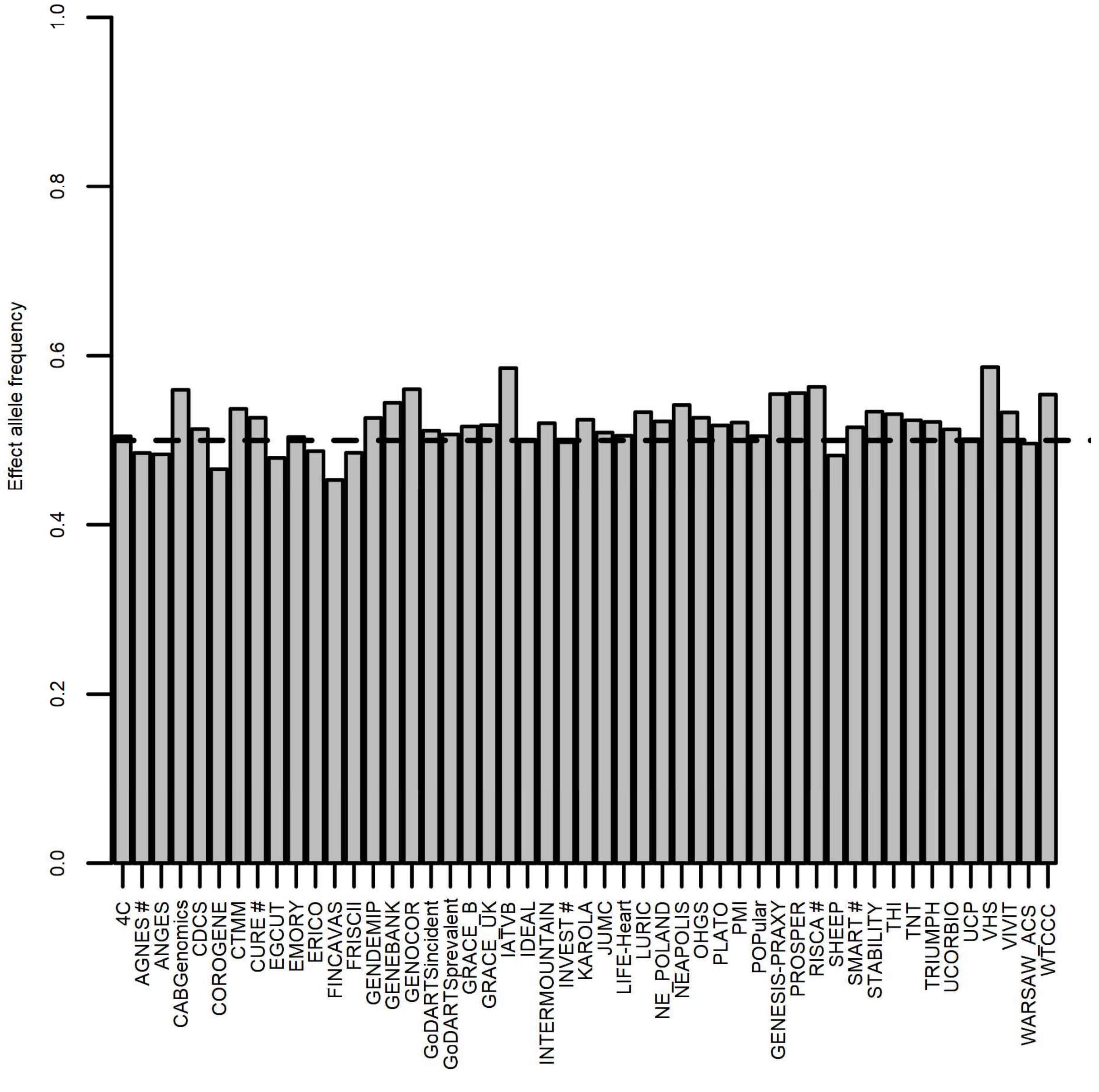

Figure 1: Study specific effect allele frequency of the GENIUS 9p21 rs1333049 variant; # indicates proxy variants rs4977574 or rs10757278. Dashed line indicates 0.50.

Figure 2

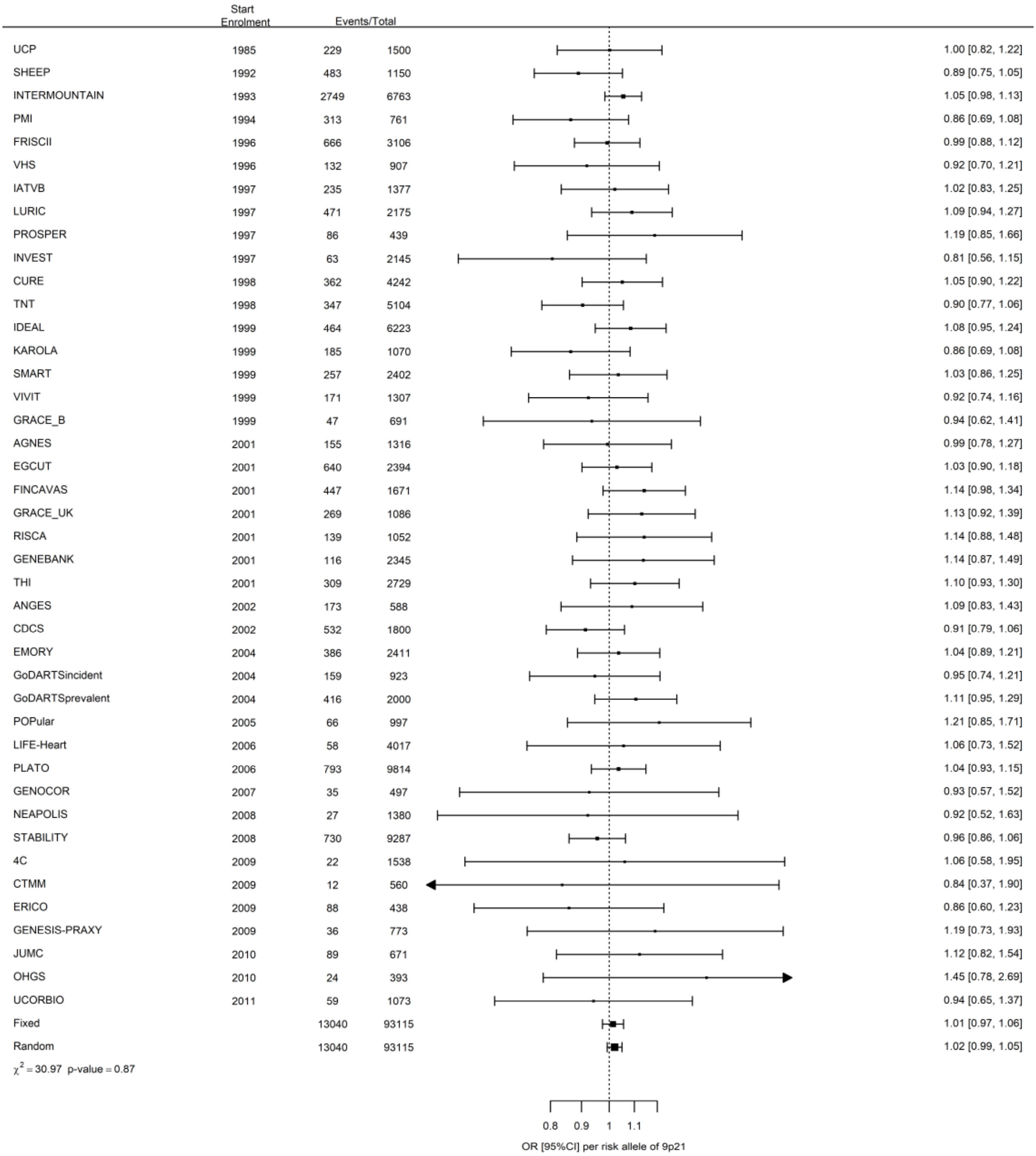

Figure 2: The study specific 9p21 associations of rs1333049 with CHD death/MI in subjects with established CHD. Estimates reflect the effect of a change in risk allele, and were adjusted for age and sex using study specific logistic regression models. Studies were ordered by start of enrollment date

Figure 3

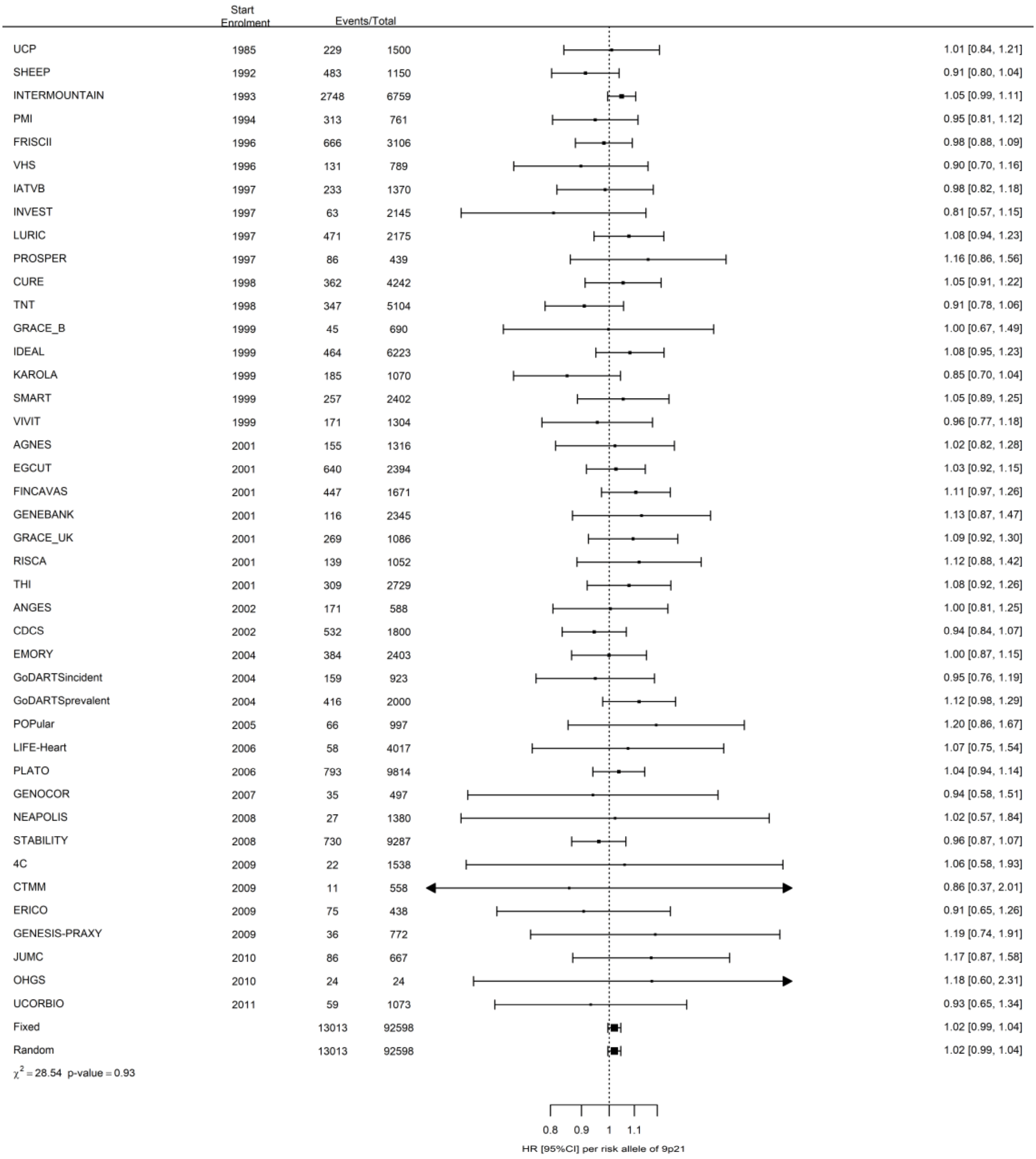

Figure 3: The study specific 9p21 associations of rs1333049 with subsequent CHD death/MI in subjects with established CHD. Estimates reflect the effect of a change in risk allele, and were adjusted for age and sex using study specific Cox proportional hazard models. Studies were ordered by start of enrollment date.

Figure 4

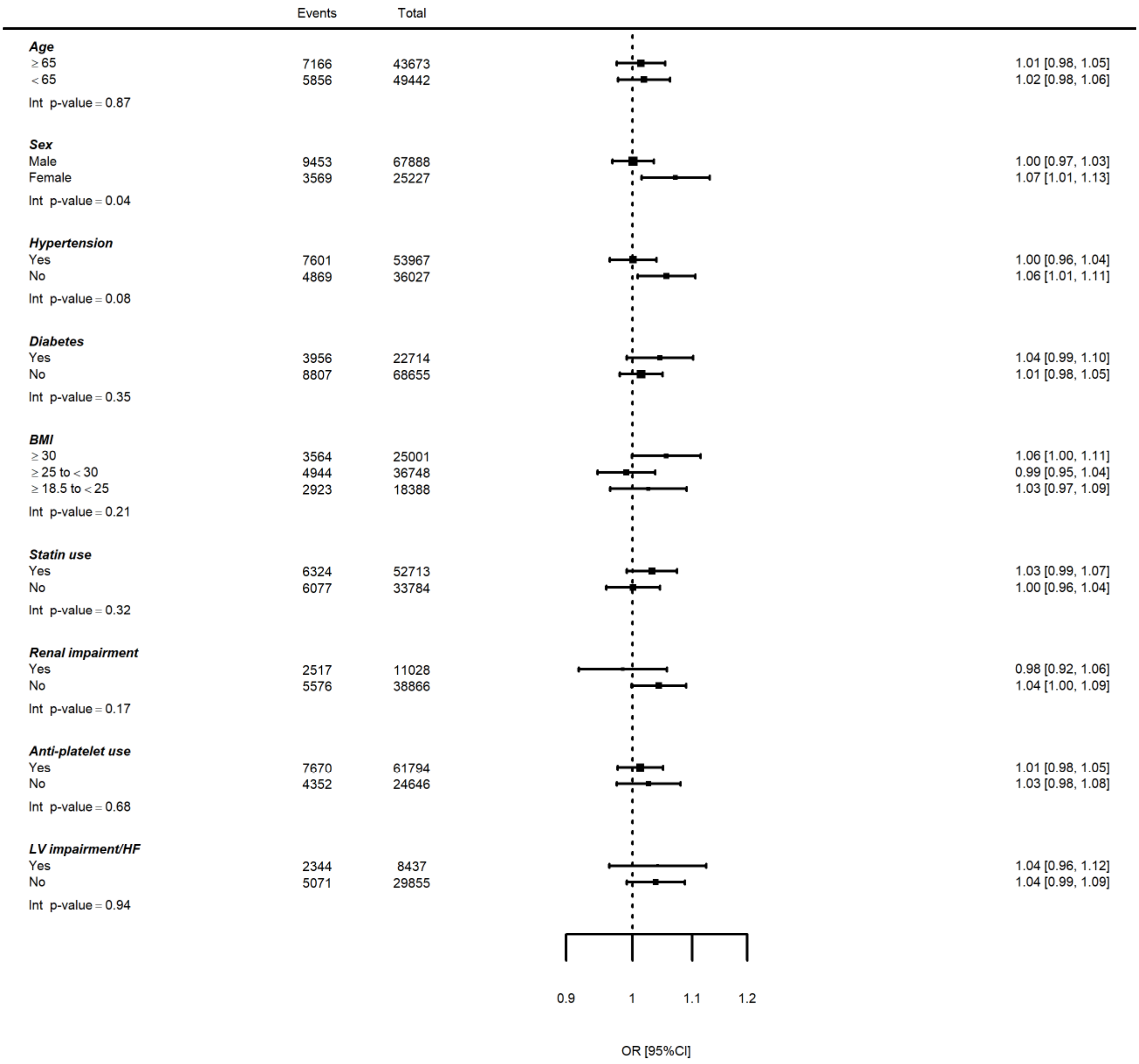

Figure 4: Subgroup effects of 9p21 on CHD death/MI among subjects with established CHD.

Figure 5

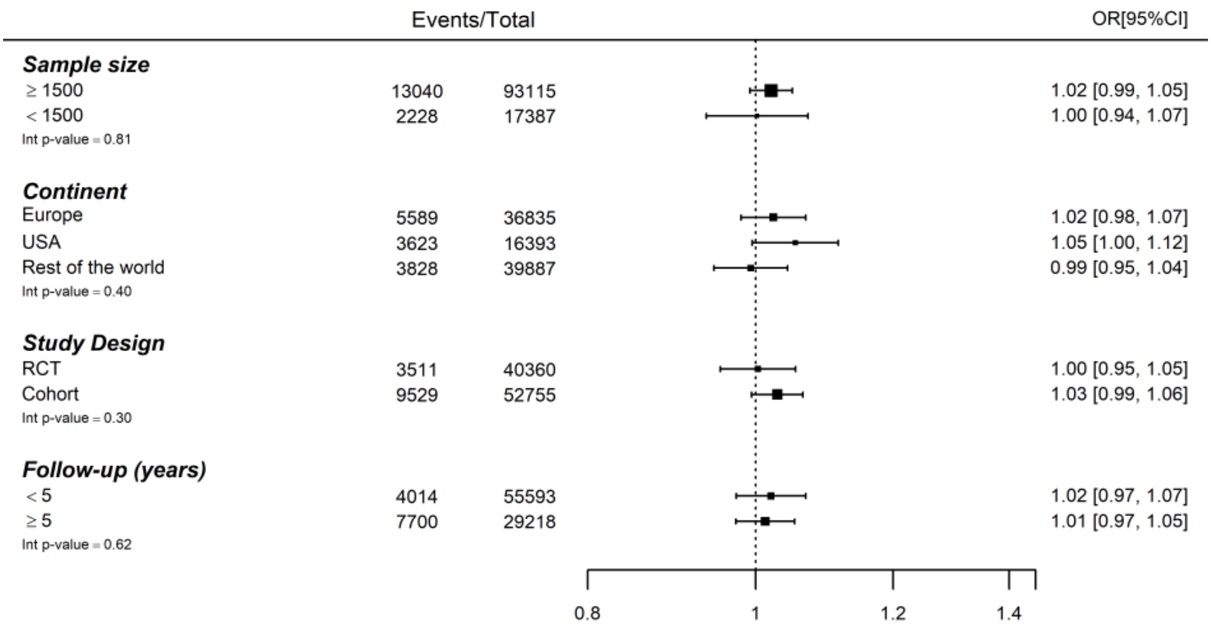

Figure 5: Study specific subgroup effects of 9p21 on CHD death/MI among subjects with established CHD.

Figure 6

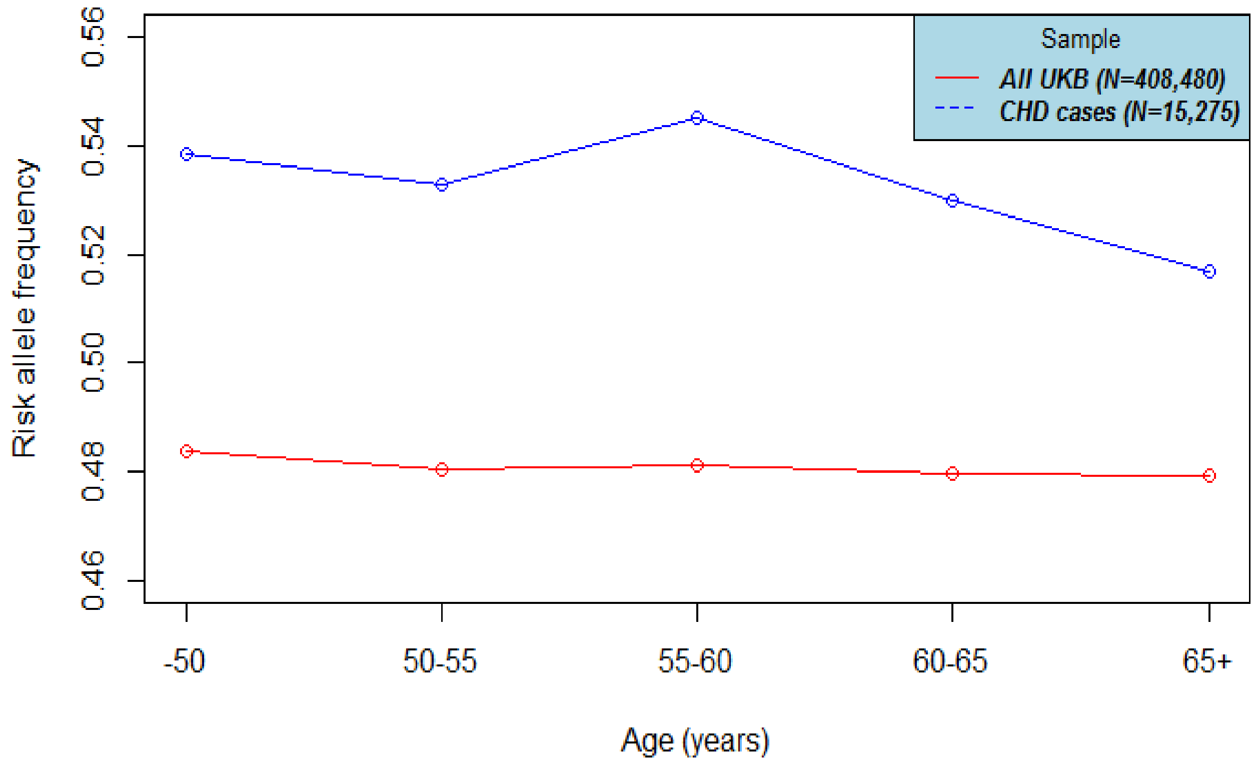

Figure 6: Risk allele frequencies by age in the UK Biobank. Risk allele frequencies by 5 year age groups, among the full UKB general population and the subset with established CHD. Risk allele for rs1333049 is C.

4 Supplemental References

1. Patel RS, et al. Subsequent Event Risk in Individuals with Established Coronary Heart Disease: Design and Rationale of the GENIUS-CHD Consortium *Circ Genom Precis Med*. 2019;12.
2. Patel RS, et al. The GENIUS-CHD consortium. *Eur Heart J*. 2015;36:2674-6.
3. Nikpay M, et al. A comprehensive 1,000 Genomes-based genome-wide association meta-analysis of coronary artery disease. *Nat Genet*. 2015;47:1121-1130.
4. Littlejohns TJ, et al. UK Biobank: opportunities for cardiovascular research. *Eur Heart J*. 2017.
5. Rothwell PM, et al. Treating individuals 3: from subgroups to individuals: general principles and the example of carotid endarterectomy. *Lancet*. 2005;365:256-65.
6. Sun X, et al. Is a subgroup effect believable? Updating criteria to evaluate the credibility of subgroup analyses. *BMJ*. 2010;340:c117.
7. Dahabreh IJ, et al. Index event bias as an explanation for the paradoxes of recurrence risk research. *JAMA*. 2011;305:822-3.
8. R: A language and environment for statistical computing [computer program]. Vienna, Austria: R Foundation for Statistical Computing,; 2018.

5 Additional Information

List of CARDIoGRAMplusC4D authors

Panos Deloukas[1,126], Stavroula Kanoni[1,126], Christina Willenborg[2,126], Martin Farrall[3,4,126], Themistocles L Assimes[5,126], John R Thompson[6,126], Erik Ingelsson[7,126], Danish Saleheen[8–10,126], Jeanette Erdmann[2,126], Benjamin A Goldstein[5], Kathleen Stirrups[1], Inke R König[11], Jean-Baptiste Cazier[4], Åsa Johansson[12], Alistair S Hall[13], Jong-Young Lee[14], Cristen J Willer[15,16], John C Chambers[17], Tõnu Esko[18,19], Lasse Folkersen[20,21], Anuj Goel[3,4], Elin Grundberg[22], Aki S Havulinna[23], Weang K Ho[10], Jemma C Hopewell[24,25], Niclas Eriksson[12], Marcus E Kleber[26,27], Kati Kristiansson[23], Per Lundmark[28], Leo-Pekka Lyytikäinen[29,30], Suzanne Rafelt[31], Dmitry Shungin[32–34], Rona J Strawbridge[20,21], Gudmar Thorleifsson[35], Emmi Tikkanen[36,37], Natalie Van Zuydam[38], Benjamin F Voight[39], Lindsay L Waite[40], Weihua Zhang[17], Andreas Ziegler[11], Devin Absher[40], David Altshuler[41–44], Anthony J Balmforth[45], Inês Barroso[1,46], Peter S Braund[31,47], Christof Burgdorf[48], , Simone Claudi-Boehm[49], David Cox[50], Maria Dimitriou[51], Ron Do[41,43], CARDIOGENICS Consortium[52], DIAGRAM Consortium[52], Alex S F Doney[38], NourEddine El Mokhtari[53], Per Eriksson[20,21], Krista Fischer[18], Pierre Fontanillas[41], Anders Franco-Cereceda[54], Bruna Gigante[55], Leif Groop[56], Stefan Gustafsson[7], Jörg Hager[57], Göran Hallmans[58], Bok-Ghee Han[14], Sarah E Hunt[1], Hyun M Kang[59], Thomas Illig[60], Thorsten Kessler[48], Joshua W Knowles[5], Genovefa Kolovou[61], Johanna Kuusisto[62], Claudia Langenberg[63], Cordelia Langford[1], Karin Leander[55], Marja-Liisa Lokki[64], Anders Lundmark[28], Mark I McCarthy[3,] 65,66, Christa Meisinger[67], Olle Melander[56], Evelin Mihailov[19], Seraya Maouche[68], Andrew D Morris[38], Martina MüllerNurasyid[69–72], MuTHER Consortium[52], Kjell Nikus[73], John F Peden[3], N William Rayner[3], Asif Rasheed[9], Silke Rosinger[74], Diana Rubin[53], Moritz P Rumpf[48], Arne Schäfer[75], Mohan Sivananthan[76,77], Ci Song[7], Alexandre F R Stewart[78,79], Sian-Tsung Tan[80], Gudmundur Thorgeirsson[81,82], C Ellen van der Schoot[83], Peter J Wagner[36,37], Wellcome Trust Case Control Consortium[52], George A Wells[78,79], Philipp S Wild[84,85], Tsun-Po Yang[1], Philippe Amouyel[86], Dominique Arveiler[87], Hanneke Basart[88], Michael Boehnke[59], Eric Boerwinkle[89], Paolo Brambilla[90], Francois Cambien[68], Adrienne L Cupples[91,92], Ulf de Faire[55], Abbas Dehghan[93], Patrick Diemert[94], Stephen E Epstein[95], Alun Evans[96], Marco M Ferrario[97], Jean Ferrières[98], Dominique Gauguier[3,99], Alan S Go[100], Alison H Goodall[31,47], Villi Gudnason[81,101], Stanley L Hazen[102], Hilma Holm[35], Carlos Iribarren[100], Yangsoo Jang[103], Mika Kähönen[104], Frank Kee[105], Hyo-Soo Kim[106], Norman Klopp[60], Wolfgang Koenig[107], Wolfgang Kratzer[108], Kari Kuulasmaa[23], Markku Laakso[62], Reijo Laaksonen[108], Ji-Young Lee[14], Lars Lind[28], Willem H Ouwehand[1,109,110], Sarah Parish[24,25], Jeong E Park[111], Nancy L Pedersen[7], Annette Peters[67,112], Thomas Quertermous[5], Daniel J Rader[113], Veikko Salomaa[23], Eric Schadt[114], Svati H Shah[115,116], Juha Sinisalo[117], Klaus Stark[118], Kari Stefansson[35,81], David- Alexandre Trégouët[68], Jarmo Virtamo[23], Lars Wallentin[12], Nicholas Wareham[63], Martina E Zimmermann[118], Markku S Nieminen[117], Christian Hengstenberg[118], Manjinder S Sandhu[1,63], Tomi Pastinen[119], Ann-Christine Syvänen[28], G Kees Hovingh[88], George Dedoussis[51], Paul W Franks[32–34,120], Terho Lehtimäki[29,30], Andres Metspalu[18,19], Pierre A Zalloua[121], Agneta Siegbahn[12], Stefan Schreiber[94], Samuli Ripatti[1,37], Stefan S Blankenberg[74], Markus Perola[23], Robert Clarke[24,25], Bernhard O Boehm[74], Christopher O'Donnell[93], Muredach P Reilly 122,126, Winfried März[26,123], Rory Collins[24,25,127], Sekar Kathiresan[41,124,125,126], Anders Hamsten[20,21,126], Jaspal S Kooner[80,126], Unnur Thorsteinsdottir[35,81,126], John Danesh9, [126], Colin N A Palmer[38,126], Robert Roberts[78,79,126], Hugh Watkins[3,4,126], Heribert Schunkert2,126 & Nilesh J Samani31,47,126

[1]Wellcome Trust Sanger Institute, Hinxton, Cambridge, UK. [2]Institut für Integrative und Experimentelle Genomik, Universität zu Lübeck, Lübeck, Germany. [3]Wellcome Trust Centre for Human Genetics, University of Oxford, Oxford, UK. [4]Cardiovascular Medicine, Radcliffe Department of Medicine, University of Oxford, Oxford, UK. [5]Department of Medicine, Stanford University School of Medicine, Stanford, California, USA. [6]Department of Health Sciences, University of Leicester, Leicester, UK.[7]Department of Medical Epidemiology and Biostatistics, Karolinska Institutet, Stockholm, Sweden.[8]Center for Non-Communicable Diseases, Karachi, Pakistan. [9]Department of

Public Health and Primary Care, University of Cambridge, Cambridge, UK. [10]Department of Medicine, University of Pennsylvania, Philadelphia, Pennsylvania, USA. [11]Institut für Medizinische Biometrie und Statistik, Universität zu Lübeck, Lübeck, Germany. [12]Uppsala Clinical Research Center, Uppsala University, Uppsala, Sweden.[13]Division of Cardiovascular and Neuronal Remodelling, Multidisciplinary Cardiovascular Research Centre, Leeds Institute of Genetics, Health and Therapeutics, University of Leeds, Leeds, UK. [14]Center for Genome Science, Korea National Institute of Health, Korea Center for Disease Control and Prevention, Yeonje-ri, Chungwon-gun, Chungcheongbuk-do, Korea. [15]Division of Cardiovascular Medicine, Department of Internal Medicine, University of Michigan, Ann Arbor, Michigan, USA.[16]Department of Human Genetics, University of Michigan, Ann Arbor, Michigan, USA. [17]Department of Epidemiology and Biostatistics, Imperial College London, London, UK. [18]Estonian Genome Center, University of Tartu, Tartu, Estonia. [19]Institute of Molecular and Cell Biology, University of Tartu, Tartu, Estonia. [20]Atherosclerosis Research Unit, Department of Medicine, Karolinska Institutet, Stockholm, Sweden. [21]Center for Molecular Medicine, Karolinska University Hospital, Stockholm, Sweden.[22]Department of Twin Research and Genetic Epidemiology, King's College London, London, UK. [23]Department of Chronic Disease Prevention, National Institute for Health and Welfare, Helsinki, Finland. [24]Clinical Trial Service Unit, University of Oxford, Oxford, UK. [25]Epidemiological Studies Unit, University of Oxford, Oxford, UK. [26]Mannheim Institute of Public Health, Social and Preventive Medicine, Medical Faculty of Mannheim, University of Heidelberg, Mannheim, Germany. [27]Ludwigshafen Risk and Cardiovascular Health (LURIC) Study, Freiburg, Germany. [28]Department of Medical Sciences, Uppsala University, Uppsala, Sweden. [29]Department of Clinical Chemistry, Fimlab Laboratories, Tampere University Hospital, Tampere, Finland. [30]Department of Clinical Chemistry, University of Tampere School of Medicine, Tampere, Finland. [31]Department of Cardiovascular Sciences, University of Leicester, Glenfield Hospital, Leicester, UK. [32]Genetic & Molecular Epidemiology Unit, Department of Clinical Sciences, Lund University Diabetes Center, Skåne University Hospital, Malmö, Sweden. [33]Department of Public Health & Clinical Medicine, Genetic Epidemiology & Clinical Research Group, Section for Medicine, Umeå University, Umeå, Sweden. [34]Department of Odontology, Umeå University, Umeå, Sweden. [35]deCODE Genetics, Reykjavik, Iceland. [36]Institute for Molecular Medicine FIMM, University of Helsinki, Helsinki, Finland. [37]Public Health Genomics Unit, National Institute for Health and Welfare, Helsinki, Finland. [38]Medical Research Institute, University of Dundee, Ninewells Hospital and Medical School, Dundee, UK. [39]Department of Pharmacology, University of Pennsylvania, Philadelphia, Pennsylvania, USA. [40]HudsonAlpha Institute for Biotechnology, Huntsville, Alabama, USA.[41]Broad Institute of Harvard and MIT, Cambridge, Massachusetts, USA. [42]Department of Molecular Biology, Massachusetts General Hospital, Boston, Massachusetts, USA. [43]Center for Human Genetic Research, Massachusetts General Hospital, Boston, Massachusetts, USA. [44]Department of Genetics, Harvard Medical School, Boston, Massachusetts, USA. [45]Division of Cardiovascular and Diabetes Research, Multidisciplinary Cardiovascular Research Centre, Leeds Institute of Genetics, Health and Therapeutics, University of Leeds, Leeds, UK. [46]University of Cambridge Metabolic Research Laboratories, Institute of Metabolic Science, Addenbrooke's Hospital, Cambridge, UK. [47]National Institute for Health Research (NIHR) Leicester Cardiovascular Biomedical Research Unit, Glenfield Hospital, Leicester, UK. [48] Deutsches Herzzentrum München, Technische Universität München, Munich, Germany. [49]Practice of Gynecology, Ulm University Medical Centre, Ulm, Germany. [50]Biotherapeutics and Bioinnovation Center, Pfizer, South San Francisco, California, USA. [51]Department of Dietetics– Nutrition, Harokopio University, Athens, Greece. [52] [53]Klinik für Innere Medizin, Kreiskrankenhaus Rendsburg, Rendsburg, Germany. [54]Cardiothoracic Surgery Unit, Department of Molecular Medicine and Surgery, Karolinska Institutet,Stockholm, Sweden. [55]Division of Cardiovascular Epidemiology, Institute of Environmental Medicine, Karolinska Institutet, Stockholm, Sweden. [56]Department of Clinical Sciences, Diabetes and Endocrinology, Lund University, University Hospital Malmö, Malmö, Sweden. [57]CEA–Genomics Institute, National Genotyping Centre, Paris, France. Commissariat à l'énergie atomique et aux energies alternatives]

[58]Department of Public Health & Clinical Medicine, Section for Nutritional Research, Umeå University, Umeå, Sweden. [59]Department of Biostatistics, Center for Statistical Genetics, University of Michigan, Ann Arbor, Michigan, USA. [60]Hannover Unified Biobank, Hannover Medical School, Hannover, Germany. [61]First Cardiology Department, Onassis Cardiac Surgery Center 356, Athens, Greece. [62]Department of Medicine, University of Eastern Finland and Kuopio University Hospital, Kuopio, Finland. [63]Medical Research Council (MRC) Epidemiology Unit, Institute of Metabolic Science, Addenbrooke's Hospital, Cambridge, UK. [64]Transplantation Laboratory, Haartman Institute, University of Helsinki, Helsinki, Finland. [65]Oxford Centre for Diabetes, Endocrinology and Metabolism, University of Oxford, Oxford, UK. [66]Oxford NIHR Biomedical Research Centre, Churchill Hospital, Oxford, UK.[67]Institute of Epidemiology II, Helmholtz Zentrum München–German Research Center for Environmental Health, Neuherberg, Germany. [68]Institut National de la Santé et la Recherche Médicale (INSERM) Unité Mixte de Recherche (UMR) S937, Institute for Cardiometabolism and Nutrition (ICAN), Pierre and Marie Curie (Paris 6) University, Paris, France. [69]Department of Medicine I, University Hospital Grosshadern, Ludwig-Maximilians-Universität, Munich, Germany. [70]Chair of Epidemiology, Institute of Medical Informatics, Biometry and Epidemiology, Ludwig-Maximilians-Universität, Munich, Germany. [71]Chair of Genetic Epidemiology, Institute of Medical Informatics, Biometry and Epidemiology, LudwigMaximilians-Universität, Munich, Germany. [72]Institute of Genetic Epidemiology, Helmholtz Zentrum München–German Research Center for Environmental Health, Neuherberg, Germany.[73]Heart Centre, Department of Cardiology, Tampere University Hospital, Tampere, Finland. [74]Division of Endocrinology and Diabetes, Department of Internal Medicine, Ulm University Medical Centre, Ulm, Germany. [75]Institut für Klinische Molekularbiologie, Christian-Albrechts Universität, Kiel, Germany. [76]Division of Epidemiology, Multidisciplinary Cardiovascular Research Centre (MCRC) University of Leeds, Leeds, UK. [77]Leeds Institute of Genetics, Health and Therapeutics, University of Leeds, Leeds, UK. [78]University of Ottawa Heart Institute, Cardiovascular Research Methods Centre Ontario, Ottawa, Ontario, Canada. [79]Ruddy Canadian Cardiovascular Genetics Centre, Ottawa, Ontario, Canada. [80]National Heart and Lung Institute (NHLI), Imperial College London, Hammersmith Hospital, London, UK. [81]Faculty of Medicine, University of Iceland, Reykjavik, Iceland. [82]Department of Medicine, Landspitali University Hospital, Reykjavik, Iceland. [83]Department of Experimental Immunohematology, Sanquin, Amsterdam, The Netherlands. [84]Center for Thrombosis and Hemostasis, University Medical Center Mainz, Johannes Gutenberg University Mainz, Mainz, Germany. [85]Department of Medicine 2, University Medical Center Mainz, Johannes Gutenberg University Mainz, Mainz, Germany. [86]Institut Pasteur de Lille, INSERM U744, Université Lille Nord de France, Lille, France. [87]Department of Epidemiology and Public Health, EA3430, University of Strasbourg, Strasbourg, France. [88]Department of Vascular Medicine, Academic Medical Center, Amsterdam, The Netherlands. [89]Human Genetics Center, University of Texas Health Science Center, Houston, Texas, USA. [90]Department of Experimental Medicine, University of Milano– Bicocca, Monza, Italy. [91]Department of Biostatistics, Boston University School of Public Health, Boston, Massachusetts, USA. [92]National Heart, Lung, and Blood Institute's Framingham Heart Study, Framingham, Massachusetts, USA. [93]Department of Epidemiology, Erasmus Medical Center, Rotterdam, The Netherlands. [94]Clinic for General and Interventional Cardiology, University Heart Center Hamburg, Hamburg, Germany. [95]Cardiovascular Research Institute, Washington Hospital Center, Washington, DC, USA. [96]Centre for Public Health, The Queen's University of Belfast, Belfast, UK. [97]Research Centre for Epidemiology and Preventive Medicine (EPIMED), Department of Clinical and Experimental Medicine, University of Insubria, Varese, Italy. [98]Department of Cardiology, Toulouse University School of Medicine, Rangueil Hospital, Toulouse, France. [99]INSERM UMR S872, Cordeliers Research Centre,Paris, France. [100]Division of Research, Kaiser Permanente Northern California, Oakland, California, USA. [101]Icelandic Heart Association, Kopavogur, Iceland. [102]Lerner Research Institute, Cleveland Clinic, Cleveland, Ohio, USA. [103]Cardiology Division, Department of Internal Medicine, Cardiovascular Genome Center, Yonsei University, Seoul, Korea. [104]Department of Clinical Physiology, Tampere University Hospital and

University of Tampere, Tampere, Finland. [105]UK Clinical Research Collaboration (UKCRC) Centre of Excellence for Public Health (Northern Ireland), Queen's University of Belfast, Belfast, UK. [106]Department of Internal Medicine, Cardiovascular Center, Seoul National University Hospital, Seoul, Korea. [107]Department of Internal Medicine II–Cardiology, Ulm University Medical Center, Ulm, Germany. [108]Science Center, Tampere University Hospital, Tampere, Finland. [109]Department of Haematology, University of Cambridge, Cambridge, UK. [110]National Health Service (NHS) Blood and Transplant, Cambridge, UK. [111]Division of Cardiology, Samsung Medical Center, Seoul, Korea. [112]Munich Heart Alliance, Munich, Germany. [113]Division of Translational Medicine and Human Genetics, Department of Medicine, Perelman School of Medicine at the University of Pennsylvania, Philadelphia, Pennsylvania, USA. [114]Institute for Genomics and Multiscale Biology, Department of Genetics and Genomic Sciences, Mount Sinai School of Medicine, New York, New York, USA. [115]Center for Human Genetics, Department of Medicine, Duke University Medical Center, Durham, North Carolina, USA. [116]Division of Cardiology, Department of Medicine, Duke University Medical Center, Durham, North Carolina, USA. [117]Division of Cardiology, Department of Medicine, Helsinki University Central Hospital (HUCH), Helsinki, Finland. [118]Klinik und Poliklinik für Innere Medizin II, Regensburg, Germany. [119]Department of Human Genetics, McGill University, Montréal, Québec, Canada. [120]Department of Nutrition, Harvard School of Public Health, Boston, Massachusetts, USA. [121]Lebanese American University, Chouran, Beirut, Lebanon. [122]Cardiovascular Institute, Perelman School of Medicine at the University of Pennsylvania, Philadelphia, Pennsylvania, USA. [123]Synlab Academy, Mannheim, Germany. [124]Cardiology Division, Center for Human Genetic Research, Massachusetts General Hospital and Harvard Medical School, Boston, Massachusetts, USA.[125]Cardiovascular Research Center, Massachusetts General Hospital and Harvard Medical School, Boston, Massachusetts, USA. [126]These authors contributed equally to this work.
