# Supplementary material for: Association of Chromosome 9p21 With Subsequent Coronary Heart Disease Events: A GENIUS-CHD Study of Individual Participant Data
Source: Circ Genom Precis Med. 2019 Apr 16;12(4):e002471. doi: 10.1161/CIRCGEN.119.002471 (PMC6625876; doi:10.1161/CIRCGEN.119.002471)
Supplement: Supplementary file 2 [file hcg-12-e002471-s002.pdf]

Association of Chromosome 9p21 with Subsequent Coronary Heart Disease Events: A GENIUS-CHD Study of Individual Participant Data

Running title: *Patel, Schmidt & Tragante, et al.; Chromosome 9p21 & Subsequent CHD events*

Riyaz S. Patel, MD[1,2]*; Amand F. Schmidt, PhD[1,3]*; Vinicius Tragante, PhD[3]*; Raymond O. McCubrey, MS[4]; Michael V. Holmes, MD, PhD[5-7]; Laurence J. Howe, PhD[1]; Kenan Direk, PhD[1]; Axel Åkerblom, MD, PhD[8,9]; Karin Leander, PhD[10]; Salim S. Virani, MD, PhD[11,12]; Karol A. Kaminski, MD, PhD[13,14]; Jochen D. Muehlschlegel, MD, MMSc[15,16]; Marie-Pierre Dubé, PhD[17,18], Hooman Allayee, PhD[19]; Peter Almgren, MSc[20]; Maris Alver, MSc[21,22]; Ekaterina V. Baranova, MSc[23]; Hassan Behlouli, PhD[24]; Bram Boeckx, PhD[25,26]; Peter S. Braund, PhD[27,28]; Lutz P. Breitling, MD[29]; Graciela Delgado, MSc[30]; Nubia E. Duarte, PhD[31]; Line Dufresne, MSc[24,32]; Niclas Eriksson, PhD[8]; Luisa Foco, PhD[33]; Crystel M. Gijsberts, MD, PhD[34]; Yan Gong, PhD[35]; Jaana Hartiala, PhD[19,36]; Mahyar Heydarpour, PhD[15,16]; Jaroslav A. Hubacek, DSc[37]; Marcus Kleber, PhD[30]; Daniel Kofink, PhD[3]; Pekka Kuukasjärvi, MD, PhD[38]; Vei-Vei Lee, MS[39]; Andreas Leiherer, PhD[40-42]; Petra A. Lenzini, MS[43]; Daniel Levin, PhD[44]; Leo-Pekka Lyytikäinen, MD[45,46]; Nicola Martinelli, MD, PhD[47]; Ute Mons, PhD[29], Christopher P. Nelson, PhD[27,28]; Kjell Nikus, MD, PhD[48,49]; Anna P. Pilbrow, PhD[50]; Rafal Ploski, MD, PhD[51]; Yan V. Sun, PhD[52,53]; Michael W.T. Tanck, PhD[54]; W.H.Wilson Tang, MD[55,56]; Stella Trompet, PhD[57,58]; Sander W. van der Laan, PhD[59]; Jessica Van Setten[60]; Ragnar O. Vilmundarson, MSc[61,62]; Chiara Viviani Anselmi, PhD[63]; Efthymia Vlachopoulou, PhD[64]; Eric Boerwinkle, PhD[65]; Carlo Briguori, MD, PhD[66]; John F. Carlquist, PhD[4,67]; Kathryn F. Carruthers, MPhil[68]; Gavino Casu, MD[69]; John Deanfield, MD[1,2]; Panos Deloukas, PhD[70,71]; Frank Dudbridge, PhD[72]; Natalie Fitzpatrick, MSc[73]; Bruna Gigante, MD, PhD[10]; Stefan James, MD, PhD[8,74]; Marja-Liisa Lokki, PhD[64]; Paulo A. Lotufo, MD, PhD[75]; Nicola Marziliano, PhD[69]; Ify R. Mordi, MD[44]; Joseph B. Muhlestein, MD[4,67]; Chris Newton Cheh, MD[76]; Jan Pitha, PhD[37]; Christoph H. Saely, MD[40,41,77]; Ayman Samman-Tahhan, MD[78]; Pratik B. Sandesara, MD[78]; Andrej Teren, MD[79,80]; Adam Timmis, MD[73,81]; Frans Van de Werf, PhD[82]; Els Wauters, PhD[83]; Arthur A.M. Wilde, MD, PhD[84,85]; Ian Ford, MD, PhD[86]; David J. Stott, MD[87]; Ale Algra, MD[88]; Maria G. Andreassi, PhD[89]; Diego Ardissino, MD[90]; Benoit J. Arsenault, PhD[91,92]; Christie M. Ballantyne, MD[12]; Thomas O. Bergmeijer, MD[93]; Connie R. Bezzina, PhD[84]; Simon C. Body, MD, MPH, MBChB[16,94]; Peter Bogaty, MD[95-97]; Gert J. de Borst, MD[98]; Hermann Brenner, MD, PhD[29]; Ralph Burkhardt, MD[80,99]; Clara Carpeggiani, MD, PhD[89]; Gianluigi Condorelli, MD, PhD[63,100]; Rhonda M. Cooper-DeHoff, PharmD[35]; Sharon Cresci, MD[43,101]; Ulf de Faire, PhD[10]; Robert N. Doughty, MD[102]; Heinz Drexel, MD[40,41,103]; James C. Engert, PhD[32,104,105]; Keith A.A. Fox, MD, PhD[106]; Domenico Girelli, MD, PhD[47]; Emil Hagström, MD, PhD[8,9]; Stanley L. Hazen, MD, PhD[55,107]; Claes Held, MD, PhD[8,9]; Harry Hemingway, MD, PhD[73]; Imo E. Hoefer, MD, PhD[108]; G. Kees Hovingh, MD, PhD[109]; Julie A. Johnson, PharmD[35,110]; Pim A. de Jong, MD[111]; J. Wouter Jukema, MD, PhD[58,112,113]; Marcin P. Kaczor, MD, PhD[114]; Mika Kähönen, PhD[115,116]; Jiri Kettner, PhD[117]; Marek Kiliszek, MD, PhD[118]; Olaf H. Klungel, PharmD, PhD[23]; Bo Lagerqvist, MD, PhD[8,74]; Diether Lambrechts, PhD[25,26]; Jari O. Laurikka, MD, PhD[119,120]; Terho Lehtimäki, PhD[45,46]; Daniel Lindholm, MD, PhD[8,9]; B.K. Mahmoodi, MD, PhD[93]; Anke H. Maitland-van der Zee, PharmD, PhD[23,121]; Ruth McPherson, MD, PhD[61,122]; Olle Melander, MD, PhD[20,123]; Andres Metspalu, MD, PhD[21,22]; Witold Pepinski, MD, PhD[124]; Oliviero Olivieri, MD[47]; Grzegorz Opolski, MD, PhD[125]; Colin N. Palmer, PhD[126]; Gerard Pasterkamp, MD, PhD[127], Carl J. Pepine, MD[110]; Alexandre C. Pereira, MD, PhD[31]; Louise Pilote, MD[24,128]; Arshed A. Quyyumi, MD[78]; A. Mark Richards, MD, PhD[50,129]; Marek Sanak, MD, PhD[114]; Markus Scholz, PhD[80,130]; Agneta Siegbahn, MD, PhD[8,131]; Juha Sinisalo, MD, PhD[132]; J. Gustav Smith, MD, PhD[133-135]; John A. Spertus, MD, MPH[136,137]; Alexandre F.R. Stewart, PhD[61,62]; Wojciech Szczeklik, MD, PhD[114]; Anna Szpakowicz, MD, PhD[14]; Jurriën M. ten Berg, MD, PhD[93]; George Thanassoulis, MD[32,24,105]; Joachim Thiery, MD[80,138]; Yolanda van der Graaf, MD[139]; Frank L.J. Visseren, MD[140]; Johannes Waltenberger[141]; CARDIoGRAMPlusC4D Consortium[142]; Pim Van der Harst, MD, PhD[143]; Jean-Claude Tardif, MD[17,18]; Naveed Sattar, PhD[87]; Chim C. Lang, MD[44]; Guillaume Pare, MD[144,145]; James M. Brophy, MD[24,128]; Jeffrey L. Anderson, MD[4,67]; Winfried März, MD[30,146,147]; Lars Wallentin, MD, PhD[8,74]; Vicky A. Cameron, PhD[50]; Benjamin D. Horne, PhD, MPH[4,148]; Nilesh J. Samani, MD, PhD[27,28]†; Aroon D. Hingorani, MD, PhD[1]†; Folkert W. Asselbergs MD, PhD[1,3,60]†

[1]Institute of Cardiovascular Science, Faculty of Population Health Science, [73]Inst of Health Informatics, Faculty of Population Health Science, Univ College London; [2]Bart's Heart Centre, St Bartholomew's Hospital; [81]Bart's Heart Centre, St Bartholomew's Hospital, London, UK; [3]Dept of Cardiology, Division Heart and Lungs, [34]Laboratory of Experimental Cardiology, [108]Dept of Clinical Chemistry and Hematology, [127]Dept of Clinical Chemistry, UMC Utrecht, Utrecht, Netherlands; [4]Intermountain Heart Inst, Intermountain Medical Ctr, Salt Lake City, UT; [5]Clinical Trial Service Unit and Epidemiological Studies Unit, Nuffield Dept of Population Health, [6]Medical Research Council Population Health Research Unit, Univ of Oxford; [7]National Inst for Health Research Oxford Biomedical Research Centre, Oxford Univ Hospital, Oxford, UK; [8]Uppsala Clinical Research Ctr; [9]Dept of Medical Sciences, Cardiology, [74]Dept of Medical Sciences, Cardiology, [131]Dept of Medical Sciences, Clinical Chemistry, Uppsala Univ, Uppsala; [10]Inst of Environmental Medicine, Karolinska Instt, Stockholm, Sweden; [11]Section of Cardiology, Michael E. DeBakey Veterans Affairs Medical Ctr, [12]Section of Cardiovascular Research, Dept of Medicine, Baylor College of Medicine, Houston, TX; [13]Dept of Population Medicine and Civilization Disease Prevention; [14]Dept of Cardiology; [124]Dept of Forensic Medicine; Medical Univ of Bialystok, Bialystok, Poland; [15]Dept of Anesthesiology, Perioperative and Pain Medicine, Brigham & Women's Hospital, [16]Harvard Medical School, Boston, MA; [17]Montreal Heart Inst; [18]Université de Montréal, Faculty of Medicine, Montreal QC, Canada; [19]Depts of Preventive Medicine and Biochemistry and Molecular Medicine, [36]Inst for Genetic Medicine, Keck School of Medicine of USC, Los Angeles, CA; [20]Dept of Clinical Sciences, Lund Univ, Malmö, Sweden; [21]Estonian

Genome Ctr, Inst of Genomics, [22]Dept of Biotechnology, Inst of Molecular and Cell Biology, Univ of Tartu, Tartu, Estonia; [23]Division of Pharmacoepidemiology and Clinical Pharmacology, [88]Dept of Neurology&Neurosurgery, Brain Centre Rudolf Magnus and Julius Ctr for Health Sciences and Primary Care, Univ Medical Ctr Utrecht, [111]Dept of Radiology, Univ Medical Ctr Utrecht, [139]Julius Ctr for Health Sciences and Primary Care, Univ Medical Ctr Utrecht, [140]Dept of Vascular Medicine, Univ Medical Ctr Utrecht & Utrecht Univ, Utrecht, Netherlands; [24]Centre for Outcomes Research & Evaluation, Research Inst of the McGill Univ Health Centre; [104]Research Inst of the McGill Univ Health Centre; [105]Division of Cardiology, Dept of Medicine, Royal Victoria Hospital, McGill Univ Health Centre; [128]Dept of Medicine, McGill Univ Health Centre, Montreal, Québec, Canada; [25]Laboratory for Translational Genetics, Dept of Human Genetics, [82]Departement of Cardiovascular Sciences, KU Leuven; [26] Laboratory for Translational Genetics, VIB Ctr for Cancer Biology, VIB, Belgium; [27]Dept of Cardiovascular Sciences, Univ of Leicester, BHF Cardiovascular Research Centre, [28]NIHR Leicester Biomedical Research Centre, Glenfield Hospital, Leicester, UK; [29] Division of Clinical Epidemiology and Aging Research, German Cancer Research Ctr (DKFZ), Heidelberg; [30] Vth Dept of Medicine, Medical Faculty Mannheim, Heidelberg Univ, Mannheim, Germany; [31] Heart Inst, Univ of Sao Paulo, Brazil; [32] Preventive and Genomic Cardiology, McGill Univ Health Ctr, Montreal, Quebec, Canada; [33] Inst for Biomedicine, Eurac Research, Affiliated Inst of the Univ of Lübeck, Bolzano, Italy; [35]Dept of Pharmacotherapy and Translational Research & Ctr for Pharmacogenomics, Univ of Florida; [110]College of Medicine, Division of Cardiovascular Medicine, Univ of Florida, Gainesville FL; [37]Centre for Experimental Medicine, Institut for Clinical and Experimental Medicine, Prague, Czech Republic; [38]Dept of Cardio-Thoracic Surgery, Finnish Cardiovascular Research Ctr - Tampere, Faculty of Medicine and Life Sciences, [46]Dept of Clinical Chemistry, Finnish Cardiovascular Research Ctr - Tampere, Faculty of Medicine and Life Sciences, [49]Dept of Cardiology, Finnish Cardiovascular Research Ctr - Tampere, Faculty of Medicine and Life Sciences, [116]Dept of Clinical Physiology, Finnish Cardiovascular Research Ctr - Tampere, Faculty of Medicine and Life Sciences, [120]Dept of Cardio-Thoracic Surgery, Finnish Cardiovascular Research Ctr - Tampere, Faculty of Medicine and Life Sciences, Univ of Tampere, Tampere, Finland; [39]Dept of Biostatistics & Epidemiology, Texas Heart Inst, Houston, TX; [40]Vorarlberg Inst for Vascular Investigation and Treatment (VIVIT), Feldkirch, Austria; [41]Private Univ of the Principality of Liechtenstein, Triesen, Liechtenstein; [42]Medical Central Laboratories, Feldkirch, Austria; [43]Dept of Genetics, Statistical Genomics Division, [101]Dept of Medicine, Cardiovascular Division Washington Univ School of Medicine, St Louis, MO; [44]Division of Molecular & Clinical Medicine, School of Medicine, Univ of Dundee, Dundee, Scotland, UK; [45]Dept of Clinical Chemistry, Fimlab Laboratories, Tampere, Finland; [47]Dept of Medicine, Univ of Verona, Italy; [48]Dept of Cardiology, Heart Ctr, [115]Dept of Clinical Physiology, [119]Dept of Cardio-Thoracic Surgery, Heart Ctr, Tampere Univ Hospital, Tampere, Finland; [50]The Christchurch Heart Inst, Univ of Otago Christchurch, Christchurch, New Zealand; [51]Dept of Medical Genetics, [125]1st Chair and Dept of Cardiology, Medical Univ of Warsaw, Warsaw, Poland; [52]Dept of Epidemiology, Emory Univ Rollins School of Public Health; [53]Dept of Biomedical Informatics, [78]Division of Cardiology, Dept of Medicine, Emory Clinical Cardiovascular Research Inst, Emory Univ School of Medicine, Atlanta, GA; [54]Clinical Epidemiology and Biostatistics, [84]AMC Heart Ctr, Clinical and Experimental Cardiology, Amsterdam Cardiovascular Sciences, Amsterdam UMC, Univ of Amsterdam, Netherlands; [55]Dept of Cellular and Molecular Medicine, Lerner Research Inst, [56]Dept of Cardiovascular Medicine, Heart and Vascular Inst & Ctr for Clinical Genomics, [107]Dept of Cardiovascular Medicine, Heart and Vascular Inst & Ctr for Microbiome and Human Health, Cleveland Clinic, OH; [57]Section of Gerontology and Geriatrics, Dept of Internal Medicine, [58]Dept of Cardiology, Leiden Univ Medical Ctr, Leiden; [59]Laboratory of Clinical Chemistry and Hematology, Division Laboratories, Pharmacy, and Biomedical Genetics, [98]Dept of Vascular Surgery, Univ Medical Ctr Utrecht, Univ Utrecht; [60]Durrer Centre of Cardiogenetic Research, ICIN-Netherlands Heart Inst, Utrecht, Netherlands; [61]Ruddy Canadian Cardiovascular Genetics Centre, Univ of Ottawa Heart Inst; [62]Dept of Biochemistry, Microbiology and Immunology, Univ of Ottawa, Ontario, Canada; [63]Dept of Cardiovascular Medicine, Humanitas Clinical and Research Ctr, Milan, Italy; [64]Transplantation Laboratory, Medicum, [132]Heart and Lung Ctr Helsinki Univ Hospital Univ of Helsinki, Helsinki, Finland; [65]Univ of Texas School of Public Health, Houston, TX; [66]Clinica Mediterranea, Naples, Italy; [67]Cardiology Division, Dept of Internal Medicine, [148]Dept of Biomedical Informatics, Univ of Utah, Salt Lake City, UT; [68]Cardiovascular Sciences, [106]Emeritus Professor of Cardiology, Univ of Edinburgh, Edinburgh; [69]ATS Sardegna, ASL 3 Nuoro; [70]William Harvey Research Inst, Barts &the London Medical School, [71]Centre for Genomic Health, Queen Mary Univ of London, London; [72]Dept of Health Sciences, Univ of Leicester, UK; [75]Centro de Pesquisa Clinica, Hospital Universitario, Universidade de Sao Paulo, Brazil; [76]Cardiovascular Research Ctr & Ctr for Human Genetic Research, Massachusetts General Hospital, Boston & Program in Medical and Population Genetics, Broad Inst, Cambridge, MA; [77]Dept of Medicine & Cardiology, Academic Teaching Hospital Feldkirch, Austria; [79]Heart Ctr Leipzig; [80]LIFE Research Ctr for Civilization Diseases, [130]Inst for Medical Informatics, Statistics and Epidemiology, Univ of Leipzig, Leipzig, Germany; [83]Respiratory Oncology Unit, Dept of Respiratory Medicine, Univ Hospitals KU Leuven, Leuven, Belgium; [85]Princess Al-Jawhara Al-Brahim Centre of Excellence in Research of Hereditary Disorders, Jeddah, Saudi Arabia;[86]Robertson Ctr for Biostatistics, [87]Inst of Cardiovascular and Medical Sciences, Univ of Glasgow, Glasgow, UK; [89]CNR Inst of Clinical Physiology, Pisa; [90]Cardiology Dept, Parma Univ Hospital, Parma, Italy; [91]Centre de recherche de l'Institut Universitaire de cardiologie et de pneumologie de Québec; [92]Dept of Medicine, Faculty of Medicine, Université Laval, Québec, Canada; [93]St. Antonius Hospital, dept. Cardiology, Nieuwegein, The Netherlands; [94]Dept of Anesthesia, Pain and Critical Care, Beth Israel Deaconess Medical Ctr, Boston, MA; [95]Service de cardiologie, Département multidisciplinaire de cardiologie, Institut universitaire de cardiologie et de pneumologie de Québec, Québec City; [96]Unité d'évaluation cardiovasculaire, Institut national d'excellence en santé et en services sociaux (INESSS), Montreal, [97]Laval Univ, Institut universitaire de cardiologie et de pneumologie de Québec, Québec City, Québec, Canada; [99]Inst of Clinical Chemistry and Laboratory Medicine, Univ Hospital Regensburg, Germany; [100]Dept of Biomedical Sciences, Humanitas Univ, Milan, Italy; [102]Heart Health Research Group, Univ of Auckland, Auckland, New Zealand; [103]Drexel Univ College of Medicine, Philadelphia PA; [109]Dept of Vascular Medicine, Academic Medical Ctr, Amsterdam; [112]Einthoven Laboratory for Experimental Vascular Medicine, LUMC, Leiden; [113]InterUniv Cardiology Inst of the Netherlands, Utrecht, Netherlands; [114]Dept of Internal Medicine, Jagiellonian Univ Medical College, Kraków, Poland; [117]Cardiology Centre, Inst for Clinical and Experimental Medicine, Prague, Czech Republic; [118]Dept of Cardiology and Internal Diseases, Military Inst of Medicine, Warsaw, Poland; [121]Dept of Respiratory Medicine, Academic Medical Ctr, Univ of Amsterdam; [122]Depts of Medicine and Biochemistry, Microbiology and Immunology, Univ of Ottawa, Ontario, Canada; [123]Dept of Internal Medicine, Skåne Univ Hospital, Malmö, Sweden; [126]Pat Macpherson Centre for Pharmacogenetics and Pharmacogenomics, Division of Molecular & Clinical Medicine, Ninewells Hospital and Medical School, Dundee; [129]Cardiovascular Research Inst, National Univ of Singapore, Singapore; [133]Dept of Cardiology, Clinical Sciences, Lund Univ & Skåne Univ Hospital; [134]Wallenberg Ctr for Molecular Medicine, Lund Univ, Lund, Sweden; [135]Lund Univ Diabetes Ctr, Lund Univ, Lund, Sweden; [136]Saint Luke's Mid America Heart Inst and the Univ of Missouri-Kansas City, [137]Saint Luke's Health System, Kansas City, MO; [138]Inst of Laboratory Medicine, Clinical Chemistry & Molecular Diagnostics, Univ Hospital, Leipzig; [141]Dept of Cardiovascular Medicine, Univ of Münster, Germany; [142]CARDIoGRAMPlusC4D; [143]Univ of Groningen, Univ Medical Ctr, Groningen, Netherlands; [144]McMaster Univ, Dept of Pathology and Molecular Medicine; [145]Population Health Research Inst, Hamilton, ON, Canada; [146]Synlab academy, Synlab Holding Deutschland GmbH, Mannheim, Germany; [147]Clinical Inst of Medical & Chemical Laboratory Diagnostics, Medical Univ of Graz, Graz, Austria

*contributed equally / † contributed equally

and Precision Medicine

Correspondence

Riyaz S. Patel
Institute of Cardiovascular Sciences
University College London
222 Euston Rd.
London, NW1 2DA
United Kingdom
Tel: 020 3549 5332
E-mail: Riyaz.Patel@ucl.ac.uk

Folkert W. Asselbergs
Department of Cardiology
Division of Heart & Lungs
University Medical Center Utrecht
3508GA, Utrecht
Netherlands
Tel: +31 887553358
E-mail: F.W.Asselbergs@umcutrecht.nl

Journal Subject Terms: Genetic, Association Studies; Coronary Artery Disease; Secondary Prevention; Epidemiology; Risk Factors

Abstract:

Background: Genetic variation at chromosome 9p21 is a recognized risk factor for coronary heart disease (CHD). However, its effect on disease progression and subsequent events is unclear, raising questions about its value for stratification of residual risk.

Methods: A variant at chromosome 9p21 (rs1333049) was tested for association with subsequent events during follow-up in 103,357 Europeans with established CHD at baseline from the GENIUS-CHD Consortium (73.1% male, mean age 62.9 years). The primary outcome, subsequent CHD death or myocardial infarction (CHD death/MI), occurred in 13,040 of the 93,115 participants with available outcome data. Effect estimates were compared to case/control risk obtained from CARDIoGRAMPlusC4D including 47,222 CHD cases and 122,264 controls free of CHD.

Results: Meta-analyses revealed no significant association between chromosome 9p21 and the primary outcome of CHD death/MI among those with established CHD at baseline (GENIUS-CHD OR 1.02; 95% CI 0.99-1.05). This contrasted with a strong association in CARDIoGRAMPlusC4D OR 1.20; 95% CI 1.18-1.22; p for interaction <0.001 compared to the GENIUS-CHD estimate. Similarly, no clear associations were identified for additional subsequent outcomes, including all-cause death, although we found a modest positive association between chromosome 9p21 and subsequent revascularization (OR 1.07; 95% CI 1.04-1.09).

Conclusions: In contrast to studies comparing individuals with CHD to disease free controls, we found no clear association between genetic variation at chromosome 9p21 and risk of subsequent acute CHD events when all individuals had CHD at baseline. However, the association with subsequent revascularization may support the postulated mechanism of chromosome 9p21 for promoting atheroma development.

American Heart Association.
Circulation: Genomic and Precision Medicine

Key words: chromosome 9p21; genetics, association studies; residual risk; prognosis; myocardial infarction; recurrent event; secondary prevention

Introduction

Using a case-control approach, a large number of common genetic variants have now been associated with coronary heart disease (CHD) through genome-wide association studies, in an effort largely led by the CARDIoGRAMPlusC4D consortium.[1] Among these variants, the chromosome 9p21 locus was the first to be discovered and the variant with the largest individual effect, and is the most widely replicated genetic risk factor for CHD.[2-4] Multiple studies including case-control and prospective cohort studies in general populations have reliably confirmed its effect on risk of CHD among otherwise healthy individuals.[5]

However, it is uncertain whether variants at the 9p21 locus also affect risk of recurrent or subsequent events, including mortality in those with established CHD. Elucidation of this hypothesis would help to better understand its mechanism and estimate its incremental value for stratification of residual risk. Prior studies have shown conflicting results, although most have been underpowered. A literature based meta-analysis indicated a null association of chromosome 9p21 variants with subsequent CHD events but was based on summary, not individual level data, with varying outcome definitions. [6, 7]

The new collaborative GENetIcs of sUbSequent Coronary Heart Disease (GENIUS-CHD) consortium, described in this issue of the journal was established to investigate genetic determinants of disease progression following an index CHD event.[8]

In this paper, we use the GENIUS-CHD resource to: (1) examine the association of variants at the 9p21 locus on risk of subsequent CHD events in individuals with established CHD; (2) compare these to the association between chromosome 9p21 and any CHD observed in the CARDIoGRAMPlusC4D consortium; and, (3) explore the potential impact on these

American Heart Association
Circulation: Genomic and Precision Medicine

estimates of biases that might affect genetic association studies of disease outcome and prognosis.

Methods

In accordance with Transparency and Openness Promotion (TOP) Guidelines, the data, analytic methods, and study materials will be made available to other researchers for purposes of reproducing the results or replicating the procedure. Participating studies received local institutional review board approval and included patients who had provided informed consent at the time of enrolment. The central analysis sites also received waivers from their local institutional review board for collating and analysing summary level data from these individual studies. Details about the GENIUS-CHD consortium and study inclusion criteria have been published separately in this issue of the journal,[8] while for this study full details about data sources, genetic variant selection, outcomes and statistical analyses are available in the Supplementary Material.

American Heart Association.
Circulation: Genomic and Precision Medicine

Results

In total, 49 studies from the GENIUS-CHD consortium contributed to the federated analysis resulting in a sample size of 103,357 individuals of European descent with established CHD and available genotype data at the 9p21 locus. Of these, 93,115 individuals had available data for the primary composite outcome of subsequent CHD death/MI, of whom 13,040 experienced these events. Contributing study details are provided in Table 1. Participant characteristics are representative for populations with established CHD with a weighted mean age of 62.9 years; 73.1% male. As expected, risk factor prevalence was high in this population, including diabetes

(24.4%), hypertension (59.1%) and current smoking (25.7%). Statin use at enrollment varied by study, ranging from 5.2%-97.3%, with a median of 61.5% (Table 1).

The rs1333049 single nucleotide polymorphism (SNP) was genotyped in 42 studies, with the remaining 7 studies using highly correlated proxies (R-squared > 0.90); rs10757278 (4 studies) or rs4977574 (3 studies) when the primary SNP was unavailable. Genotyping details are provided in Supplementary Table 1. For rs1333049, the average risk allele frequency across the participating studies was 0.518 ranging from 0.453 to 0.587 (Supplementary Figure 1).

From CARDIOGRAMplusC4D, after excluding 6 cohorts which had contributed data to both consortia, data were available for association with chromosome 9p21 from 41 studies, including 47,222 cases with CHD and 122,264 controls free of any CHD.

American Heart Association.
Circulation: Genomic and Precision Medicine

Power to detect different effect sizes, including the effect size identified in CARDIoGRAMplusC4D, using a two-sided alpha of 0.05, are provided in Supplementary Table 2.

Chromosome 9p21 Association with Subsequent CHD Events

Study-specific results for the association between chromosome 9p21 and risk of the primary outcome of CHD death or myocardial infarction among individuals with established CHD at baseline, adjusted for age and sex are presented in Supplementary Figure 2.

The per-allele odds ratio for the primary outcome during follow-up was 1.02 (95%CI 0.99-1.05). The effect estimate again for the primary outcome, based on a time to event analysis and using a Cox regression model, was also similar with a HR of 1.02 (95%CI 0.99-1.04, Supplementary Figure 3).

In contrast, a meta-analysis of CARDIOGRAMIplusC4D data (excluding studies also contributing data to GENIUS-CHD), revealed a per-allele odds ratio for a CHD event similar to

that reported previously (OR 1.20; 95% CI 1.18-1.22). There was evidence of statistical heterogeneity between the estimates (Interaction p<0.001), Figure 1**.**

Subgroup Analyses

We found minimal evidence for heterogeneity in effect estimates when stratifying by CHD subtype at baseline (interaction p-value 0.801), with no clear evidence for an effect of chromosome 9p21 genetic variation on subsequent CHD death or MI in individuals enrolled with ACS (OR 1.02; 95% CI 0.97-1.06), those with coronary artery disease with a prior MI (OR 1.01; 95% CI 0.96-1.05) and those with coronary artery disease without prior MI (OR 1.01; 95% CI 0.95-1.08, Figure 1).

We further examined the effect of chromosome 9p21 on the primary outcome in pre-specified subgroup analyses. We noted a borderline nominally significant interaction with sex, suggesting a greater risk among women with the chromosome 9p21 risk allele, for subsequent CHD death/MI (interaction p value = 0.04), while non-significant trends were noted for greater risk in those without hypertension (p value = 0.08) or without renal impairment (p value = 0.17). There were minimal differences in effect estimates by other patient level characteristics including age and diabetes, or according to statin or anti-platelet use or LV impairment at baseline (Supplementary Figure 4).

American Heart Association
Circulation: Genomic and Precision Medicine

Similarly, when stratified by study level features, we observed minimal evidence for heterogeneity in effect estimates by study size, geographical region, study design or length of follow up (Supplementary Figure 5). However, when ordered by date of first enrollment, there was no evidence for variation in effect by time of enrollment (Supplementary Figure 2).

Secondary outcomes

We additionally examined the association between chromosome 9p21 and other subsequent events available for this analysis within the GENIUS-CHD Consortium, listed in Supplementary Table 3, with summary estimates provided in Figure 2. Of note, the per-allele effect of risk variants at chromosome 9p21 on subsequent revascularization during follow up was 1.07 (95% CI 1.04-1.09). The effect on the composite outcome of any CVD, which includes revascularization, was also significant at 1.04 (95% CI 1.02-1.07). However, there was no clear evidence of association for the remaining secondary outcomes, with only a marginal trend to protection for both subsequent heart failure (OR 0.97; 95% CI 0.93-1.01) and CVD death (OR 0.97; 95% CI 0.94-1.01), as shown in Figure 2.

Selection Bias

American Heart Association.
Circulation: Genomic and Precision Medicine

To explore the potential for index event bias we looked for differences in associations between chromosome 9p21 and known cardiovascular risk factors in the UKB, among the subset of participants with established CHD, compared to the full UKB cohort (Supplementary Table 4). While there were differences between the groups in the prevalence or values of the tested risk factors, we did not find clear evidence to indicate a distortion in associations between chromosome 9p21 and age, blood pressure, diabetes or smoking. There was, however, a small difference for BMI, with a greater statistical association between the chromosome 9p21 risk allele and lower BMI identified in those with established CHD than in the general population (nominal interaction p value 0.02, Supplementary Table 4).

We also observed that the chromosome 9p21 risk allele frequency in those surviving with CHD, both in UKB (0.529) and in GENIUS-CHD (0.518, Supplementary Figure 1), was higher than the general population in the UKB (0.481) and European reference populations from the

1000 Genomes (Phase 3),[9] (0.472). This difference in frequency confirms the association of chromosome 9p21 with CHD and also indicated absence of a crude survival bias with loss of large numbers of risk allele carriers to fatal events prior to entry into CHD cohorts. We did however observe a trend to an age association in those with established CHD as well as the general population in the UKB, with lower chromosome 9p21 risk allele frequencies with advancing age, relative to younger carriers (Supplementary Figure 6).

Discussion

In this study, we examined the effect of genetic variation at the chromosome 9p21 locus on risk of subsequent events in 103,357 individuals with established CHD using the newly formed GENIUS-CHD consortium.[8] We found that: (1) in contrast to the known strong association with CHD observed in CARDIoGRAMPlusC4D, there was a markedly attenuated and non-significant association with subsequent CHD events in GENIUS-CHD; (2) effect estimates in GENIUS-CHD were broadly consistent in stratified analyses based on features related to study design, patient characteristics and type of index CHD event and (3) exploratory analyses suggested that selection biases were unlikely to explain the discrepancy. However, we did find evidence of an association between these variants and a secondary outcome of future revascularization events. Our findings, taken together with those from others, support the view that chromosome 9p21 promotes CHD through progressive stable atheroma rather than through development of an unstable phenotype.

The chromosome 9p21 locus is the most widely-replicated genetic risk locus for CHD identified to date, with an estimated 15-35% increased risk in carriers of the variant allele in prospective population and case-control studies.[5] However, studies examining the effect on

American Heart Association
Circulation: Genomic and Precision Medicine

subsequent CHD events in people with known CHD at baseline have reported conflicting results.[10-14] Our group previously examined this in a literature-based meta-analysis, based on 15 studies with median sample size of 1,750 individuals, accruing 25,163 cases of established CHD, and reported no clear evidence of an effect of variants at chromosome 9p21 on the risk of subsequent events.[15] An analysis by the CHARGE consortium of 2,953 MI survivors also reported no association with subsequent mortality.[7] However, the limited size of most prior studies and the limitations of literature meta-analyses indicate that many possible explanations, including errors in risk allele coding and selection biases, could not be adequately explored, precluding meaningful interpretations for any mechanistic or clinical implications.

The emergence of the GENIUS-CHD Consortium has now permitted a robust evaluation of the role of chromosome 9p21 in subsequent CHD event risk, revealing a clear lack of association with a common composite coronary endpoint. This is in marked contrast to findings from studies comparing cases to CHD-free controls, as confirmed through meta-analysis of CARDIoGRAMPlusC4D data. Furthermore, we were able to add to previous findings by showing that the type of CHD at baseline, whether acute coronary syndrome or stable CHD with or without prior MI, does not alter this association. We also interrogated several widely-proposed explanations that could account for our findings through pre-specified subgroup analyses, and confirmed that most of these, specifically older age, medication use at baseline (statin or antiplatelet), study size or follow up duration, did not appreciably alter the association findings. Our finding of a possible interaction with sex, warrants further investigation, but should be considered hypothesis-generating given the borderline evidence of an interaction.

Selection bias (i.e., index event bias or collider-stratification bias) could potentially explain reversed or attenuated associations in disease progression studies like this, operating by

American Heart Association
Circulation: Genomic and Precision Medicine

inducing relationships between (otherwise independent) risk factors through the selection of individuals with disease.[16, 17] Specifically, individuals surviving a first event consequent on exposure to a particularly strong risk factor may have lower levels of exposure to other individually weaker, independent risk factors, which can then attenuate the association of the risk factor of interest with subsequent events. However, the distribution of common risk factors by chromosome 9p21 genotype did not differ when compared between the general population and the subset with CHD in the UKB, using interaction tests. The only exception was for BMI, a potentially differential association with chromosome 9p21 in those with CHD compared to the general population was noted. However, the effect size was small in both populations and on its own is unlikely to indicate presence of substantial index event bias.

American Heart Association. Circulation: Genomic and Precision Medicine

Selection bias may also theoretically occur by focussing on subjects surviving a first event, where chromosome 9p21 risk allele carriers at risk of fatal CHD events are lost prior to enrollment into CHD cohorts, thereby diluting the future impact of the variant on subsequent CHD events. In this scenario, we would expect a lower risk-allele frequency in those surviving CHD and entering CHD cohorts, but we found no evidence for this. Among those with CHD in the UKB, and among the whole UKB cohort, we did find a progressive loss of risk allele carriers with increasing age, consistent with prior findings of a greater association with CHD, among younger individuals in case-control studies.[5] Given patients with CHD are generally older, it is possible that a subtle survival bias may still be influencing our findings, although all analyses were adjusted for age. However, based on simulation modelling, sample size and projected SNP effect size, we and others have previously estimated that selection biases are only minimally operating in this context and would be unlikely to account for our observed findings. [18, 19] Although our findings potentially argue against important selection biases in the analysis for the

primary outcome, they are relatively insensitive assessments and may not fully elucidate such biases.

Possible biological explanations could also exist for our findings. Pathological studies indicate differences between chronic stable atherosclerotic plaques that cause ischemia through progressive vessel occlusion and vulnerable plaques with thin caps, prone to sudden plaque rupture, unheralded MI and coronary deaths.[20] In a seminal study dissecting the phenotype of CHD, a lack of effect for chromosome 9p21 and MI was noted, when both cases and controls had underlying atherosclerosis.[21] Our group and others have in parallel shown that chromosome 9p21 robustly associates with atherosclerotic phenotypes,[22] while functional studies have also implicated this region with molecular activity that drives atheroma.[23] Furthermore, in this study we show that the only outcome positively associated with chromosome 9p21 is incident revascularization, perhaps reflecting more severe atherosclerosis burden. Collectively these data support the concept that chromosome 9p21 promotes progressive atheroma formation and does not confer risk via plaque rupture.

American Heart Association. Circulation: Genomic and Precision Medicine

In this context, it is worth noting that chromosome 9p21 associates more robustly with CHD in case-control studies than in prospective cohort studies.[7] The difference, as proposed by others, could hypothetically be accounted for by incidence–prevalence bias, with chromosome 9p21 carriers more likely to *survive* a CHD event and thus be *over* represented among CHD cases (the opposite to survival bias described above).[7] This becomes more likely as stated above if chromosome 9p21 drives a more progressive and stable atheroma phenotype. If this holds true then among survivors with established CHD, one might expect that chromosome 9p21 carriers could hold a small favourable advantage over those who experience CHD in its absence, due instead to other more dangerous or vulnerable characteristics, and despite undergoing more

subsequent revascularization, these chromosome 9p21 carriers do not experience more dangerous or fatal events.

These findings have important implications. Clinically, they indicate that a degree of caution should be applied when considering or evaluating patients for chromosome 9p21 in order to predict disease progression or residual risk. They also highlight the need to appreciate important biases that may inflate or attenuate association findings in the setting of subsequent events for individuals with established disease. Mechanistically, these findings support existing and emerging efforts seeking to elucidate the mechanism of the most robust genetic discovery for CHD in recent decades.

There are important limitations to consider. First, among individuals in GENIUS with established CHD, the timing of the first CHD event or age of onset was often unknown so we could not account for this variable in our analyses. However, the lack of association in the ACS studies, which had documented timing of the first event, suggests this did not impact the findings. Second, we had limited information on whether subsequent revascularization events were late staged procedures, which would count as part of the index CHD event or unplanned and symptom driven and thereby a true subsequent event, which may have diluted the effect estimate. Third, while we did not observe a specific interaction for statin or aspirin use, we cannot rule out an effect of combined or additional medication usage attenuating the association signal, given the high prevalence of secondary prevention drug use in this setting compared to general population cohorts. Fourth, our analyses were restricted to participants of European descent as most of the included studies only recruited these individuals and so we were markedly underpowered to explore associations in other ethnic groups. Unfortunately, this remains a wider problem of genetic research and global efforts are ongoing to address this imbalance.

American Heart Association.
Circulation: Genomic and Precision Medicine

Finally, variability of follow-up duration across studies is an analytical challenge and could have impacted our findings, through misclassification. However, a sensitivity analysis stratifying on the follow-up duration of individual studies (<5 or 5≥ years) revealed minimal evidence (P=0.62) of heterogeneity in effect estimates (Supplemental Figure 5), suggesting that this is unlikely to have influenced our findings significantly as effect estimates were concordant across studies with different lengths of follow-up. Our major strengths however include the size of the study and the large number and types of subsequent events and an effort to examine for selection biases. We also sought to mitigate potential miscoding of the risk allele, given rs1333049 is a palindromic SNP, and also the risk allele C changes from being a minor allele in population cohorts to the major allele in CHD cohorts. Finally, this analysis benefitted from the collective expertise and input of over 170 investigators and analysts, many of whom have previously reported on chromosome 9p21.

American Heart Association.
Circulation: Genomic and Precision Medicine

In conclusion, using the newly formed GENIUS-CHD consortium, we demonstrate that variation at chromosome 9p21 shows no clear association with risk of subsequent CHD events when all individuals have established CHD at baseline. This is in marked contrast to prior case-control studies examining odds of CHD presence compared to disease-free controls. We could not account for the attenuation of effect in terms of selection biases or subgroup effects. However, we did find a greater risk for incident revascularization in those with established CHD and although residual bias may be at play, our findings collectively support the view that chromosome 9p21 promotes CHD through progressive stable atheroma rather than through development of an unstable phenotype.

Acknowledgments: The GENIUS CHD collaborators would like to express their immense gratitude to all patients who participated in each of the individual studies as well as the many

personnel who helped with recruitment, collection, curation, management and processing of the samples and data. We also thank the CARDIoGRAMPlusC4D steering committee for providing the summary data after excluding the cohorts already in GENIUS-CHD.

Sources of Funding: The funder(s) of the study had no role in study design, data collection, data analysis, data interpretation, or writing of the report. Within GENIUS-CHD, all participating investigators and sponsors who contributed data and analyses are acknowledged irrespective of academic or industry affiliations.

Specific funding statements: Dr Patel is funded by a British Heart Foundation Intermediate Fellowship (FS/14/76/30933). This research was also supported by the National Institute for Health Research University College London Hospitals Biomedical Research Centre.; Dr Schmidt is funded by BHF grant PG/18/5033837 ; Dr Holmes works in a unit that receives funding from the UK Medical Research Council and is supported by a British Heart Foundation Intermediate Clinical Research Fellowship (FS/18/23/33512) and the National Institute for Health Research Oxford Biomedical Research Centre.; The AGNES study was supported by research grants from the Netherlands Heart Foundation (2001D019, 2003T302, 2007B202 and the PREDICT project (CVON 2012-10)), the Leducq Foundation (grant 05-CVD) and the Center for Translational Molecular Medicine (CTMM COHFAR). ; The Cleveland Clinic Genebank Study was supported in part by NIH grants R0133169, R01ES021801, R01MD010358, and R01ES025786, R01HL103866, R01DK106000, R01HL126827, P20HL113452., P01HL098055, P01HL076491, and R01HL103931; The Clinical Cohorts in Coronary disease Collaboration (4C) study was supported in part by NIHR and Barts Charity; The Corogene study was supported by grants from Aarno Koskelo Foundation, Helsinki University Central Hospi-tal special government funds (EVO #TYH7215, #TKK2012005, #TYH2012209, #TYH2014312), and Finnish Foundation for Cardiovascular research ; CABGenomics was supported by Stanton Shernan, C. David Collard, Amanda A. Fox/R01 HL 098601 NHLBI; The CDCS and PMI studies were funded by the Health Research Council and Heart Foundation of New Zealand; Dr Samman-Tahnan is supported by the National Institutes of Health/ National Institutes of Aging grant AG051633; Dr Sandesara is supported by the Abraham J. & Phyllis Katz Foundation (Atlanta, GA, USA); The Emory Cardiovascular Biobank is supported by National Institutes of Health (NIH) grants 5P01HL101398-02, 1P20HL113451-01, 1R56HL126558-01, 1RF1AG051633-01, R01

American Heart Association
Circulation: Genomic and Precision Medicine

NS064162-01, R01 HL89650-01, HL095479-01, 1U10HL110302-01, 1DP3DK094346-01, 2P01HL086773-06A1; This Estonian Biobank was funded by EU H2020 grant 692145, Estonian Research Council Grant IUT20-60, IUT24-6, PUT1660, PUT735 and European Union through the European Regional Development Fund Project No.2014-2020.4.01.15-0012 GENTRANSMED, NIH – GIANT, ERA-CVD grant Detectin-HF and 2R01DK075787-06A1.; GENESIS-PRAXY is funded by the Canadian Institutes of Health Research and Heart and Stroke Foundations of Alberta, NWT & Nunavut, British Columbia and Yukon, Nova Scotia, Ontario, and Quebec (HSFC); The GENDEMIP study was supported by Project (MH, Czech Republic) No. 00023001 (ICEM, Prague); GoDARTS was funded by the Wellcome Trust (072960/Z/03/Z, 084726/Z/08/Z, 084727/Z/08/Z, 085475/Z/08/Z, 085475/B/08/Z) and as part of the EU IMI-SUMMIT programme. C.N.A.P. has received grant funding from the Wellcome Trust to develop the GoDARTS cohort. ; Dr Mordi is supported by an NHS Education of Scotland/Chief Scientist Office Postdoctoral Clinical Lectureship (PCL 17/07); the GENECOR stuidy was supported in part by the Italian Ministry of Research's Fund for Basic Research (FIRB 2005); GRACE UK was supported in part by an Educational Grant from Sanofi Aventis; Award from Chief Scientist Office, Scotland; INVEST-GENES was supported by the National Institute of Health Pharmacogenomics Research Network grant U01-GM074492, NIH R01 HL074730, University of Florida Opportunity Fund, BASF Pharma and Abbott Laboratories. ; IATVB was supported by Epidemiologia e Genetica della Morte Improvvisa in Sardegna; The KAROLA study has received financial support by the German Ministry of Education and Research (01GD9820/0 and 01ER0814), by the Willy-Robert-Pitzer Foundation, and by the Waldburg-Zeil Clinics Isny.; The KRAKOW GENIUS Study was supported by a grant from the Polish Ministry of Science and Higher Education, no. NN402083939 and the National Science Centre, no. 2013/09/B/NZ5/00770; LIFE-Heart was funded by the Leipzig Research Center for Civilization Diseases (LIFE). LIFE is an organizational unit affiliated to the Medical Faculty of the University of Leipzig. LIFE is funded by means of the European Union, by the European Regional Development Fund (ERDF) and by funds of the Free State of Saxony within the framework of the excellence initiative.; The LURIC study was supported by the 7th Framework Program (AtheroRemo, grant agreement number 201668 and RiskyCAD, grant agreement number 305739) of the European Union; The NEAPOLIS CAMPANIA study was suppported by European Research Council Advanced Grant (CardioEpigen, #294609);Italian Ministry of Health

American Heart Association. Circulation: Genomic and Precision Medicine

(PE-2013-02356818);Italian Ministry of Education, University and Research (2015583WMX) ; The North East Poland Myocardial Infarction Study was supported by grant N N 402 529139 from the National Science Center (Poland); Dr Vilmundarson is supported by a graduate fellowship of the University of Ottawa Heart Institute; OHGS was funded in part by a Heart and Stroke Foundation grant; Dr Stott was supported in part by an investgator initiated grant from Bristol Myers Squibb USA; The PROSPER study was supported by an investigator initiated grant obtained from Bristol-Myers Squibb. Prof. Dr. J. W. Jukema is an Established Clinical Investigator of the Netherlands Heart Foundation (grant 2001 D 032). Support for genotyping was provided by the seventh framework program of the European commission (grant 223004) and by the Netherlands Genomics Initiative (Netherlands Consortium for Healthy Aging grant 050-060-810). ; The RISCA study ws supported in part by FRSQ, HSFC, Merck Frost Canada, Pfizer Canada; The SHEEP study was supported by grants from the Swedish Council for Work Life and Social Research, and the Stockholm County Council. ; The TNT trial was sponsored by Pfizer who granted access to data, Genotyping of the samples was funded in part by grants from Genome Canada and Genome Quebec and the Canadian Institutes of Health Research (CIHR).; Dr Arsenault holds a junior scholar award from the Fonds de recherche du Quebec- Sante (FRQS); Dr. Cresci is supported, in part, by the National Institutes of Health (Cresci R01 NR013396). The TRIUMPH study was sponsored by the National Institutes of Health: Washington University School of Medicine SCCOR Grant P50 HL077113; The UCP studies were funded by the Netherlands Heart Foundation and the Dutch Top Institute Pharma Mondriaan Project; The Verona Heart Study was supported by the Cariverona Foundation; Veneto Region; Italian Ministry of Education, University, and Research (MIUR); LURM (Laboratorio Universitario di Ricerca Medica) Research Center, University of Verona; The Warsaw ACS Registry is supported by grant N R13 0001 06 from The National Centre for Research and Development (NCBiR), Statutory Grant from Medical University of Warsaw; Dr Nelson is funded by the British Heart Foundation; Prof. Samani is funded by the British Heart Foundation and is a NIHR Senior Investigator. Prof Hingorani is a NIHR Senior Investigator; Prof Asselbergs is supported by UCL Hospitals NIHR Biomedical Research Centre, EU/EFPIA Innovative Medicines Initiative 2 Joint Undertaking BigData@Heart grant n° 116074, the European Union’s Horizon 2020 research and innovation programme under the ERA-NET Co-

American Heart Association. Circulation: Genomic and Precision Medicine

fund action N°01KL1802 (Druggable-MI-gene) jointly funded by the Dutch Heart Foundation and Netherlands Organization for Health Research and Development (ZonMw).

Disclosures: Dr Patel has received speaker fees and honoraria from Amgen, Sanofi and Bayer and research grant funding from Regeneron; Dr Holmes has collaborated with Boehringer Ingelheim in research, and in accordance with the policy of The Clinical Trial Service Unit and Epidemiological Studies Unit (University of Oxford), did not accept any personal payment; Dr Akerblom has received institutional research grant and speakers fee from AstraZeneca, institutional research grant from Roche Diagnostics.; Dr James has received grants from AstraZeneca, The Medicines Company, Swedish heart and lung foundation, Swedish research council, Janssen; personal fees from Bayer.; Dr Hagstrom declares being an expert committee member, lecture fees, and institutional research grant from Sanofi, and Amgen; institutional research grants from AstraZeneca, and GlaxoSmithKline; expert committee member and lecture fees NovoNordisk and Behringer.; Dr Held declares institutional research grant, advisory board member and speaker's bureau from AstraZeneca; institutional research grants from Bristol-Myers Squibb Merck & Co, GlaxoSmithKline, Roche Diagnostics. Advisory board for Bayer and Boehringer Ingelheim; Dr Lindholm has received institutional research grants from AstraZeneca, and GlaxoSmithKline; Speaker fees from AstraZeneca, Speaker fees from AstraZeneca; Dr Siegbahn has received institutional research grants from AstraZeneca, Boehringer Ingelheim, Bristol-Myers Squibb/Pfizer, Roche Diagnostics, GlaxoSmithKline.; Dr. J.M. ten Berg reports receiving fees for board membership from AstraZeneca, consulting fees from AstraZeneca, Eli Lilly, and Merck, and lecture fees from Daiichi Sankyo and Eli Lilly, AstraZeneca, Sanofi and Accumetrics; Prof Wallentin reports institutional research grants, consultancy fees, lecture fees, and travel support from Bristol-Myers Squibb/Pfizer, AstraZeneca, GlaxoSmithKline, Boehringer Ingelheim; institutional research grants from Merck & Co, Roche Diagnostics; consultancy fees from Abbott; and holds a patent EP2047275B1 licensed to Roche Diagnostics, and a patent US8951742B2 licensed to Roche Diagnostics; Dr Claes reports lecture fees, and an institutional research grant from Sanofi, and Amgen; institutional research grants from AstraZeneca, and GlaxoSmithKline; and lecture fees from NovoNordisk and AstraZeneca. Prof Asselbergs has received research funding from Regeneron, Pfizer, Sanofi.

American Heart Association.
Circulation: Genomic and Precision Medicine

References:

1. Nikpay M, et al. A comprehensive 1,000 Genomes-based genome-wide association meta-analysis of coronary artery disease. *Nat Genet*. 2015;47:1121-1130.

2. Helgadottir A, et al. A common variant on chromosome 9p21 affects the risk of myocardial infarction. *Science*. 2007;316:1491-3.

3. McPherson R, et al. A common allele on chromosome 9 associated with coronary heart disease. *Science*. 2007;316:1488-91.

4. Samani NJ, et al. Genomewide association analysis of coronary artery disease. *N Engl J Med*. 2007;357:443-53.

5. Palomaki GE, et al. Association between 9p21 genomic markers and heart disease: a meta-analysis. *JAMA*. 2010;303:648-56.

6. Patel RS, et al. Genetic variants at chromosome 9p21 and risk of first versus subsequent coronary heart disease events: a systematic review and meta-analysis. *J Am Coll Cardiol*. 2014;63:2234-45.

7. Dehghan A, et al. Genome-Wide Association Study for Incident Myocardial Infarction and Coronary Heart Disease in Prospective Cohort Studies: The CHARGE Consortium. *PLoS One*. 2016;11:e0144997.

8. Patel RS, et al. Subsequent Event Risk in Individuals with Established Coronary Heart Disease: Design and Rationale of the GENIUS-CHD Consortium *Circ Genom Precis Med*. 2019;12.

9. Genomes Project C, et al. A global reference for human genetic variation. *Nature*. 2015;526:68-74.

10. Ellis KL, et al. A common variant at chromosome 9P21.3 is associated with age of onset of coronary disease but not subsequent mortality. *Circ Cardiovasc Genet*. 2010;3:286-93.

11. Gong Y, et al. Chromosome 9p21 haplotypes and prognosis in white and black patients with coronary artery disease. *Circ Cardiovasc Genet*. 2011;4:169-78.

12. Horne BD, et al. Association of variation in the chromosome 9p21 locus with myocardial infarction versus chronic coronary artery disease. *Circ Cardiovasc Genet*. 2008;1:85-92.

13. Virani SS, et al. Chromosome 9p21 single nucleotide polymorphisms are not associated with recurrent myocardial infarction in patients with established coronary artery disease. *Circ J*. 2012;76:950-6.

American Heart Association. Circulation: Genomic and Precision Medicine

14. Wauters E, et al. Influence of 23 coronary artery disease variants on recurrent myocardial infarction or cardiac death: the GRACE Genetics Study. *Eur Heart J*. 2013;34:993-1001.

15. Patel RS, et al. Genetic variants at chromosome 9p21 and risk of first versus subsequent coronary heart disease events: A systematic review and meta-analysis. *J Am Coll Cardiol*. 2014.

16. Dahabreh IJ, et al. Index event bias as an explanation for the paradoxes of recurrence risk research. *JAMA*. 2011;305:822-3.

17. Cole SR, et al. Illustrating bias due to conditioning on a collider. *Int J Epidemiol*. 2010;39:417-20.

18. Anderson CD, et al. The effect of survival bias on case-control genetic association studies of highly lethal diseases. *Circ Cardiovasc Genet*. 2011;4:188-96.

19. Hu YJ, et al. Impact of Selection Bias on Estimation of Subsequent Event Risk. *Circ Cardiovasc Genet*. 2017;10.

20. Falk E, et al. Update on acute coronary syndromes: the pathologists' view. *Eur Heart J*. 2013;34:719-28.

21. Reilly MP, et al. Identification of ADAMTS7 as a novel locus for coronary atherosclerosis and association of ABO with myocardial infarction in the presence of coronary atherosclerosis: two genome-wide association studies. *Lancet*. 2011;377:383-92.

22. Chan K, et al. Association between the chromosome 9p21 locus and angiographic coronary artery disease burden: a collaborative meta-analysis. *J Am Coll Cardiol*. 2013;61:957-70.

23. Visel A, et al. Targeted deletion of the 9p21 non-coding coronary artery disease risk interval in mice. *Nature*. 2010;464:409-12.

American Heart Association. Circulation: Genomic and Precision Medicine

Table 1: Overview of studies contributing to chromosome 9p21 analysis and participant characteristics

Alias	Cohort	Total N genotyped	Study Design	CHD Type	Male (%)	Age (SD)	BMI (SD)	Diabetes (%)	Smoking (%)	Systolic BP (SD)	Total Cholesterol (SD)	Statin use (%)	Creatitine (SD)	Prior Revasc (%)	Prior MI (%)	PubMED ID
4C	Clinical Cohorts in Coronary disease Collaboration (4C)	1538	Cohort	CAD	62.1	62.2 (11.95)	30.2 (5.67)	23.4	-	133.9 (23.7)	4.69 (1.10)	26.4	99.3 (83.2)	22.6	15.5	-
AGNES	Arrhythmia Genetics in The Netherlands	1316	Cohort	ACS	79.3	57.7 (10.81)	26.5 (3.87)	7.6	59.3	-	5.28 (1.04)	9.8	-	-	-	20622880
ANGES	Angiography and Genes Study	588	Cohort	Mixed	65.5	64.1 (9.55)	28.1 (4.36)	30.8	14.7	-	4.84 (0.84)	69.4	83.0 (32.0)	42.4	24.7	21640993
ATVB	Italian Atherosclerosis, Thrombosis and Vascular Biology Group	1465	Cohort	ACS	90.4	40.0 (4.40)	26.8 (4.07)	8.4	78.7	132.3 (20.6)	5.76 (1.39)	56.2	-	-	-	21757122
CABGenomics	Coronary Artery Bypass Genomics	1542	Cohort	Mixed	80.1	64.7 (10.08)	29.7 (5.71)	10.1	11.2	-	4.21 (0.95)	75.2	-	-	42.8	25649697
CDCS	Coronary Disease Cohort Study	1800	Cohort	ACS	71.5	67.5 (11.96)	27.3 (4.66)	15.4	5.8	129.2 (21.6)	5.00 (1.09)	46.5	100.5 (40.0)	26.9	30.3	20400779
COROGENE	Corogene Study	1489	Cohort	ACS	70.9	64.7 (11.87)	27.6 (4.84)	18.2	34.4	-	4.63 (0.99)	5.2	84.0 (44.3)	-	-	21642350
CTMM	Circulating Cells	605	Cohort	Mixed	68.9	63.0 (9.83)	27.6 (4.45)	20.7	20.7	135.4 (19.1)	4.43 (1.05)	-	86.4 (34.9)	-	30.1	23975238
CURE	Cure-Genetics Study	4242	RCT	ACS	59.3	64.7 (10.99)	27.9 (4.44)	19.9	22.6	135.7 (21.9)	-	-	93.0 (33.9)	13.9	31.8	11102254
EGCUT	Estonian Biobank	2408	Cohort	CAD	51.0	67.1 (10.88)	28.9 (5.16)	18.7	19.2	135.6 (18.0)	5.64 (1.17)	27.3	-	15.7	36.0	24518929
EMORY	Emory Cardiovascular Biobank	2411	Cohort	Mixed	70.1	64.5 (11.06)	-	30.7	9.8	-	4.49 (1.02)	76.0	99.0 (45.1)	61.7	27.9	20729229
ERICO	Estratégia de Registro de Insuficiência Coronariana	438	Cohort	ACS	55.5	63.8 (13.36)	27.0 (5.06)	39.1	31.0	99.2 (38.4)	-	23.8	-	11.3	25.9	23644870
FINCAVAS	Finnish Cardiovascular Study	1671	Cohort	Mixed	69.4	60.9 (11.03)	27.8 (4.35)	18.4	24.3	140.2 (22.1)	4.74 (0.90)	57.3	90.8 (66.8)	32.6	39.0	16515696
FRISCII	FRISCII Study	3106	RCT	ACS	69.4	66.2 (9.80)	26.8 (3.87)	12.7	27.1	143.3 (22.4)	5.80 (1.12)	12.3	90.6 (18.8)	12.1	27.2	10475181
GENDEMIP	GENetic DEtermination of Myocardial Infarction in Prague	1267	Cohort	ACS	75.8	56.4 (8.63)	28.6 (4.68)	18.8	60.8	137.0 (20.8)	5.51 (1.17)	16.6	-	29.7	41.6	23249639
GENEBANK	Cleveland Clinic Genebank Study	2345	Cohort	Mixed	74.3	61.5 (11.06)	29.4 (5.44)	11.8	16.8	132.7 (21.1)	4.46 (0.93)	71.8	-	65.3	56.1	21475195
GENESIS-PRAXY	GENdEr and Sex determInantS of cardiovascular disease: From bench to beyond-Premature Acute Coronary Syndrome (GENESIS-PRAXY)	784	Cohort	ACS	69.2	48.3 (5.62)	-	13.8	44.2	139.5 (26.5)	4.85 (1.18)	93.1	75.9 (19.7)	11.3	11.4	22607849
GENOCOR	Genetic Mapping for Assessment of Cardiovascular Risk	497	Cohort	Mixed	86.7	65.2 (8.45)	-	13.3	64.4	129.5 (20.3)	4.70 (0.92)	72.1	94.8 (27.2)	13.7	63.2	22717531
GoDARTS incident	Genetics of Diabetes Audit and Research in Tayside Scotland (I)	1003	Cohort	CAD	62.0	71.1 (10.62)	29.7 (5.64)	77.9	-	126.7 (NA)	4.62 (1.02)	50.8	108.0 (64.6)	0.2	1.3	-
GoDARTS prevalent	Genetics of Diabetes Audit and Research in Tayside Scotland (P)	2000	Cohort	CAD	66.5	69.1 (9.20)	30.3 (5.43)	77.8	14.9	136.2 (19.7)	4.37 (0.83)	66.8	101.6 (34.5)	31.4	48.9	-
GRACE_B	Global Registry of Acute Coronary Events - Belgium	699	Cohort	ACS	75.4	65.7 (12.01)	27.0 (4.35)	81.3	49.9	138.8 (25.3)	5.33 (1.19)	79.3	102.8 (61.9)	-	80.1	20231156
GRACE_UK	Global Registry of Acute Coronary Events - UK	1086	Cohort	ACS	69.1	64.4 (12.04)	28.0 (5.15)	15.0	69.5	137.8 (27.1)	5.19 (1.29)	16.8	105.0 (40.0)	20.4	32.0	20231156
IDEAL	Incremental Decrease in End Points Through Aggressive lipid Lowering (IDEAL)	6223	RCT	ACS	81.8	61.2 (9.32)	27.4 (3.80)	11.4	20.3	136.8 (19.8)	5.03 (0.98)	76.9	100.1 (16.7)	41.3	-	16287954
INTERMOUNTAIN	Intermountain Heart Collaborative Study	6763	Cohort	Mixed	66.7	61.2 (11.05)	29.5 (6.08)	20.3	10.2	141.8 (24.4)	4.96 (1.12)	38.7	99.6 (66.6)	-	6.6	20691829
INVEST	International Verapamil SR Trandolopril Study Genetic SubstudyNVEST-GENES	2145	RCT	CAD	56.6	68.7 (9.38)	-	23.9	12.8	148.6 (18.1)	-	52.8	-	47.6	-	21372283
JUMC	Krakow-GENIUS-CHD	704	Cohort	Mixed	71.6	68.3 (10.25)	26.3 (4.46)	36.9	27.5	148.1 (23.8)	5.02 (1.06)	88.3	89.9 (37.5)	50.1	39.7	28444280
KAROLA	Karola Study	1147	Cohort	Mixed	84.6	58.6 (8.13)	27.0 (3.26)	18.5	32.4	119.9 (15.5)	4.46 (0.84)	77.4	82.4 (26.5)	42.8	21.6	24829374
LIFE-Heart	Leipzig (LIFE) Heart Study	4330	Cohort	Mixed	75.5	64.0 (11.15)	29.0 (4.68)	34.4	29.0	138.3 (21.8)	5.24 (1.18)	38.9	87.3 (34.6)	-	0.1	22216169
LURIC	The LUdwigshafen RIsk and Cardiovascular Health Study	2175	Cohort	Mixed	76.5	63.8 (9.85)	27.5 (3.89)	44.3	23.9	142.3 (24.1)	4.94 (0.99)	58.9	88.7 (38.5)	48.7	57.4	11258203
NE_POLAND	North East Poland Myocardial Infarction Study	603	Cohort	ACS	75.0	62.4 (11.86)	24.8 (3.79)	22.2	48.1	138.9 (27.4)	5.04 (1.05)	80.7	91.6 (36.3)	1.3	10.6	26086777
NEAPOLIS	Neapolis Campania Italia	1380	Cohort	Mixed	74.4	67.6 (10.49)	28.0 (4.18)	43.0	26.8	129.4 (14.2)	4.57 (1.02)	82.5	101.0 (68.1)	41.5	40.8	24262617
OHGS	Ottawa Heart Genomics Study	393	Cohort	Mixed	73.0	65.3 (11.07)	28.6 (5.00)	6.9	19.5	131.9 (19.0)	5.53 (1.03)	92.4	89.8 (21.1)	28.2	21.9	-

PLATO	The Study of Platelet Inhibition and Patient Outcomes	9814	RCT	ACS	69.5	62.6 (10.95)	28.2 (4.51)	22.8	35.2	135.6 (21.8)	5.42 (1.23)	79.7	85.6 (26.3)	15.1	20.6	19332184
PMI	Post Myocardial Infarction Study	783	Cohort	ACS	78.3	62.7 (10.29)	26.5 (3.82)	12.0	28.2	117.0 (15.6)	5.98 (1.19)	46.0	87.8 (27.8)	-	17.2	12771003
POPular	The POPular study	997	RCT	ACS	74.3	63.8 (10.40)	-	18.9	27.5	145.0 (22.1)	4.25 (0.64)	80.7	92.7 (26.8)	33.1	43.7	26542508
PROSPER	Prospective Study of Pravastatin in the Elderly at Risk	439	RCT	CAD	69.9	75.4 (3.31)	26.4 (3.87)	10.3	16.2	150.0 (21.6)	5.55 (0.83)	-	109.4 (23.2)	26.0	85.9	10569329
RISCA	Recurrance and Inflammation in the Acute Coronary Syndromes Study	1052	Cohort	ACS	75.9	61.9 (11.40)	27.2 (4.43)	19.8	30.4	-	-	46.6	100.6 (28.6)	28.2	27.9	18549920
SHEEP	Stockholm Heart Epidemiology Program (SHEEP)	1150	Cohort	ACS	70.7	59.3 (7.21)	26.8 (4.02)	18.2	50.0	131.8 (20.6)	6.28 (1.16)	-	-	-	-	17667644
SMART	Second Manifestations of Arterial Disease	2485	Cohort	Mixed	82.2	60.2 (9.26)	27.3 (3.63)	16.6	24.4	137.4 (19.8)	4.73 (0.96)	75.7	92.3 (22.7)	-	43.6	10468526
STABILITY	Stabilization of Atherosclerotic Plaque by Initiation of Darapladib Therapy trial	9287	RCT	Mixed	82.0	64.7 (9.10)	29.9 (4.97)	38.4	21.4	131.7 (16.1)	-	97.3	-	74.6	58.6	24678955
THI	Texgen	2729	Cohort	ACS	75.3	63.6 (10.62)	29.6 (5.59)	30.5	21.3	-	-	57.1	-	21.5	16.7	21414601
TNT	Treating to New Targets	5104	RCT	CAD	81.3	61.3 (8.73)	28.6 (4.59)	14.8	13.4	130.9 (16.8)	4.51 (0.61)	70.3	104.8 (17.3)	-	57.3	15755765
TRIUMPH	Translational Research Investigating Underlying Disparities in Acute Myocardial Infarction Patient's Health Status	1974	Cohort	ACS	72.4	59.8 (12.04)	29.5 (5.96)	28.6	37.4	117.8 (18.3)	-	89.0	112.5 (76.7)	27.0	18.3	21772003
UCORBIO	Utrecht Coronary Biobank	1073	Cohort	Mixed	75.6	65.4 (10.26)	27.2 (4.34)	21.5	23.1	-	4.76 (1.18)	64.0	91.9 (42.9)	-	28.9	-
UCP	Utrecht Cardiovascular Pharmacogenetics Study	1500	Cohort	Mixed	75.4	64.1 (9.96)	-	-	-	153.4 (21.4)	5.50 (1.10)	27.1	94.7 (24.8)	-	-	25652526
VHS	Verona Heart Study	907	Cohort	CAD	80.9	61.3 (9.78)	26.9 (3.57)	18.5	69.2	-	5.37 (1.10)	47.1	96.7 (32.2)	17.4	59.6	10984565
VIVIT	Vorarlberg Institute for Vascular Investigation and Treatment Study	1318	Cohort	CAD	73.1	64.5 (10.44)	27.4 (4.14)	30.6	18.9	137.2 (19.2)	5.45 (1.14)	49.7	88.4 (32.7)	21.1	31.0	24265174
WARSAW ACS	Warsaw ACS Genetic Registry	669	Cohort	ACS	74.5	63.6 (11.72)	28.1 (4.72)	21.9	42.0	127.8 (22.6)	4.99 (1.07)	-	93.5 (41.4)	-	18.6	-
WTCCC	WTCCC CAD Study	1924	Cohort	Mixed	79.3	60.0 (8.13)	27.6 (4.20)	11.7	12.8	143.6 (22.0)	5.28 (0.98)	71.6	-	67.1	72.0	17634449

Overview of studies contributing to chromosome 9p21 analysis and participant characteristics; alias denotes the abbreviated name of each study used in figures and tables; ACS = acute coronary syndrome, CAD = coronary artery disease; PubMed IDs are provided for individual study descriptions; mean (standard deviation) with proportions (%) are provided unless otherwise stated.

American Heart Association
Circulation: Genomic and Precision Medicine

Figure Legends:

Figure 1: Association between chromosome 9p21 and subsequent CHD events in all participants with baseline CHD (GENIUS-CHD) compared to association in CHD cases and CHD-free controls (CARDIoGRAMPlusC4D)

For the CARDIoGRAMPlusC4D meta-analysis estimate, 6 studies [LURIC, LIFE-Heart, GoDARTS, OHGS, PROSPER, WTCCC] were excluded as they were also included in GENIUS-CHD.

Estimates for GENIUS-CHD are also presented by subtype of CHD at baseline including acute coronary syndrome (ACS), stable coronary artery disease without prior MI (CAD/no MI) and Stable CAD with prior MI (CAD/MI). All estimates were adjusted for age and sex.

American Heart Association.
Circulation: Genomic and Precision Medicine

Figure 2: Association between chromosome 9p21 and secondary outcomes in participants with baseline CHD, within GENIUS-CHD. All meta-analysis estimates were adjusted for age and sex.

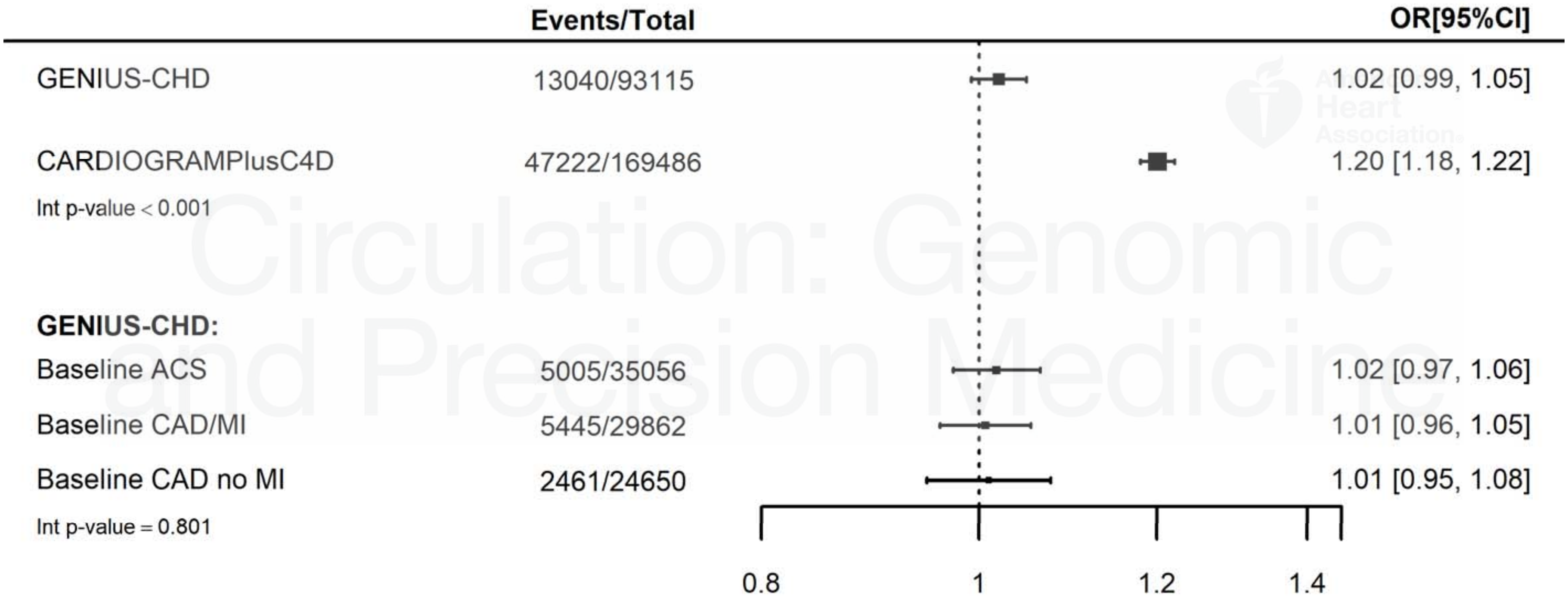

Circulation: Genomic and Precision Medicine
American Heart Association

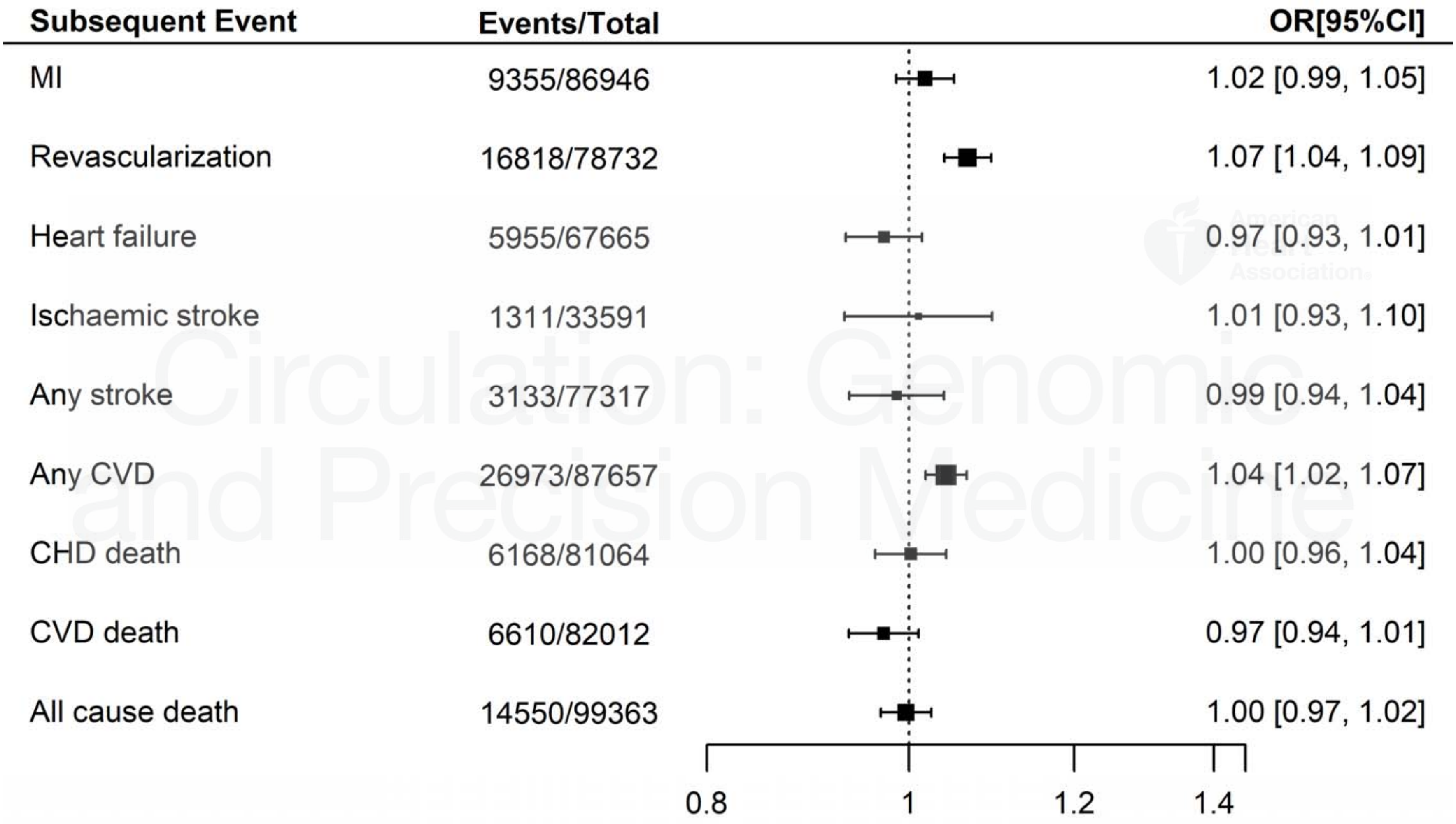

Circulation: Genomic and Precision Medicine
American Heart Association
